# IL‐33/ST2 Signaling Sustains Hepato‐Intestinal Homeostasis by Orchestrating Vascular Surveillance and Immune Regulatory Circuits During Experimental *Trypanosoma cruzi* Infection

**DOI:** 10.1096/fj.202600565RR

**Published:** 2026-07-05

**Authors:** Marcelo Eduardo Cardozo, Tatyane Martins Cirilo, José Bryan da Rocha Rihs, Jorge Lucas Nascimento Souza, Isabela de Brito Duval, Ana Rafaela Antunes‐Porto, Luisa Vitor Braga do Amaral, Fernando Bento Rodrigues Oliveira, Mayra Fernanda Ricci, Laura Lis de Oliveira Santos, Lívia Fernanda Dias Santana, Luiza Pinheiro Silva, Chiara Cássia Oliveira Amorim, Gabriela Gomes Monteiro Lemos, Getulío Mota e Silva Junior, Izabela da Silva Oliveira, Marina Possa dos Reys, Ana Laura Grossi de Oliveira, Geovanni Dantas Cassali, Luisa Mourão Dias Magalhães, Lilian Lacerda Bueno, Fabiana Simão Machado, Ricardo Toshio Fujiwara

**Affiliations:** ^1^ Laboratory of Immunobiology and Parasite Control, Institute of Biological Sciences Universidade Federal de Minas Gerais Belo Horizonte Brazil; ^2^ Post‐Graduation Program in Health Sciences: Infectious Diseases and Tropical Medicine, Faculdade de Medicina Universidade Federal de Minas Gerais Belo Horizonte Brazil; ^3^ Laboratory of Immunoregulation of Infectious Diseases, Institute of Biological Sciences Universidade Federal de Minas Gerais Belo Horizonte Brazil; ^4^ Laboratory of Comparative Pathology, Institute of Biological Sciences, Institute of Biological Sciences Universidade Federal de Minas Gerais Belo Horizonte Brazil; ^5^ Laboratory of Interactions in ImmunoParasitology, Institute of Biological Sciences, Institute of Biological Sciences Universidade Federal de Minas Gerais Belo Horizonte Brazil; ^6^ Institute of Research in Mucosa and Skin (INCT Mucosa and Skin), Universidade Federal de Minas Gerais Belo Horizonte Brazil

**Keywords:** CD44, Chagas disease, immunopathology, patrolling monocytes, vascular homeostasis

## Abstract

Chagas disease, caused by *Trypanosoma cruzi*, is characterized by a complex interplay between parasite persistence and host‐driven immunopathology. Although the IL‐33/ST2 axis is known to regulate type 2 immunity and tissue repair, its contribution to tissue homeostasis during chronic infection remains poorly understood. Using ST2‐deficient (ST2^−/−^) and wild‐type BALB/c mice followed for up to 100 days postinfection, we investigated the role of IL‐33/ST2 signaling in coordinating hepato–intestinal response and systemic immunity. ST2 deficiency induced coordinated systemic disturbances, including platelet expansion and hyperalbuminemia. At the tissue level, loss of ST2 exacerbated hepatic inflammation and fibrotic remodeling. In the colon, ST2^−/−^ mice displayed increased nitric oxide production and enhanced parasite clearance, but developed marked structural alterations. Our findings suggest that IL‐33/ST2 signaling is associated with regulatory programs. ST2 deficiency was associated with a reduction in patrolling monocytes, suggesting impaired homeostatic endothelial monitoring. This profile also coincided with inflammatory monocyte‐derived dendritic cell differentiation and lowered macrophage regulatory activity. This altered profile was associated with amplified IL‐12–driven Th1 and cytotoxic T‐cell responses while impairing IL‐10–associated regulatory niches, resulting in multiorgan inflammation. These findings suggest that IL‐33/ST2 signaling may contribute to immunoregulatory balance during *T. cruzi* infection and identify this axis as a candidate pathway for future mechanistic and therapeutic investigation.

## Graphical abstract

Impairment of hepato‐intestinal homeostasis triggered by ST2 deficiency during *T. cruzi* infection. Within the splenic compartment, immune dysregulation is marked by a robust pro‐inflammatory polarization of the T‐cell landscape. In the liver, ST2 deficiency is associated with hepatocellular injury, increased parasite burden, and hyperalbuminemia. Conversely, the colonic compartment exhibits a compensatory response characterized by increased mucus production and NO, limiting local parasite burden alongside increased vascular permeability. Collectively, these disrupted circuits transition into progressive multiorgan immunopathology during infection.

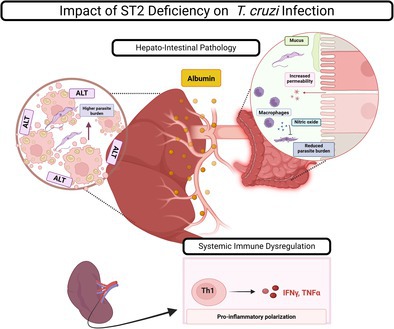

## 1 Introduction

Chagas disease (CD) remains an important neglected tropical disease, affecting approximately 10.5 million people worldwide and imposing a substantial burden through chronic cardiac and gastrointestinal complications [1]. *Trypanosoma cruzi* infection elicits a robust immune response that is indispensable for parasite control; nonetheless, clinical progression ultimately depends on the host's ability to balance effector with tissue‐protective regulatory mechanisms [2, 3]. Understanding how immunity is shaped to achieve equilibrium between protection and pathology is therefore central to the development of rational immunomodulatory strategies.

Type 1 inflammatory immunity, characterized by interferon‐gamma (IFN‐ γ) and tumor necrosis factor (TNF) production, plays a critical role in restricting *T. cruzi* replication during acute infection [4, 5, 6]. Sustained activation of these pathways, however, is associated with progressive organ‐specific pathology and fibrotic remodeling. Effective disease resolution requires robust regulatory mechanisms, including FOXP3‐dependent programs and IL‐10–mediated resolution. Disruption of these regulatory circuits, evidenced by impaired regulatory T cell function [7], and a dominant inflammatory TNFR1/IL‐10R signaling profile [8], characterizes severe clinical forms of Chagas disease, highlighting the critical role of immune regulation in preventing tissue damage.

Interleukin‐33 (IL‐33), a member of the IL‐1 cytokine family, functions both as an alarmin released upon tissue injury and as an immunomodulatory cytokine, signaling through its receptor ST2 [9, 10]. Beyond its classical association with type 2 immunity, IL‐33/ST2 signaling has emerged as a broader regulator of tissue repair, vascular integrity, and immune homeostasis [11, 12, 13]. During *T. cruzi* infection, evidence suggests that IL‐33 enhances early parasite control in the skeletal muscle while limiting tissue damage and dysfunction [14, 15]. However, the contribution of IL‐33/ST2 signaling to systemic immunity and extra‐muscular tissue homeostasis remains incompletely understood as the infection progresses from acute damage to chronic persistence.

In the present study, we aimed to investigate the role of the IL‐33/ST2 axis as a central integrator of the host immune response and tissue homeostasis during *T. cruzi* infection. We hypothesized that disruption of this signaling pathway uncouples effector immunity from regulatory mechanisms, leading to exacerbated pathology. To test this, we employed an integrative approach, combining tissue pathology, biochemical markers, and high‐dimensional flow cytometry, to evaluate the impact of ST2 deficiency on systemic inflammation and extra‐muscular homeostasis. Our study sought to characterize how disruption of IL‐33/ST2 signaling is associated with alterations in systemic immunity and tissue homeostasis during *T. cruzi* infection.

## 2 Materials and Methods

### 2.1 Ethics Statement

All experimental procedures involving animals were conducted in accordance with the guidelines of the Brazilian College of Animal Experimentation (COBEA). All study protocols were approved by the Ethics Committee on Animal Use (CEUA) of the Federal University of Minas Gerais (UFMG, Brazil) under protocol number #12/2024. All efforts were made to minimize animal suffering throughout the study.

### 2.2 Animals

Female wild‐type (WT) and ST2‐deficient (ST2^−/−^) BALB/c mice, aged 8–10 weeks, were used in all experiments. WT BALB/c mice were obtained from the Central Animal Facility of UFMG. ST2^−/−^ mice on a BALB/c genetic background were originally provided by Dr. João Santana da Silva (University of São Paulo, Brazil) and subsequently bred and maintained at the animal facility of the Department of Parasitology, Institute of Biological Sciences (ICB), UFMG. Mice were housed under a 12‐h light/dark cycle, at a controlled temperature (24°C ± 1°C), with ad libitum access to filtered water and commercial chow (Nuvilab Cr‐1, Nuvital Nutrients, Brazil).

### 2.3 *Trypanosoma cruzi* Experimental Infection

Blood trypomastigotes of the *T. cruzi* Y strain (DTU TcII), maintained by serial passage in Swiss mice, were used throughout the study. WT and ST2^−/−^ BALB/c mice were infected intraperitoneally with 1000 blood trypomastigotes suspended in sterile phosphate‐buffered saline (PBS). Infection was confirmed by parasitemia using 5 μL of tail vein blood, as described by Brener [16].

### 2.4 Experimental Design

To investigate the contribution of the IL‐33/ST2 signaling pathway to hepato‐intestinal pathology during *T. cruzi* infection, WT and ST2^−/−^ mice were followed for up to 100 days postinfection (dpi). Clinical parameters, hematological indices, biochemical markers, and immunopathological alterations were evaluated longitudinally. Animals were euthanized at predefined time points: day 0 (noninfected controls), day 7 (early acute phase), day 20 (peak acute phase), and day 100 (chronic phase). Each experimental group consisted of 5–6 animals per time point. Euthanasia was performed by ketamine (130 mg/kg) and xylazine (8.5 mg/kg) overdose.

### 2.5 Clinical Evaluation and Scoring

Animals were monitored from day 5 postinfection until day 100 dpi. Disease progression was monitored using body weight variation and the Rapid Murine Coma and Behavior Scale (RMCBs), which assesses ten parameters including gait, balance, motor performance, and grooming. Each parameter was scored from 0 (normal function) to 2 (severe impairment) [17].

Stool consistency was evaluated using a modified scoring system adapted from murine colitis models: normal formed pellets (score 0), soft/loose stools (score 2), mucoid stools (score 4), or diarrhea with liquid consistency (score 6) [18]. Fresh fecal samples were assessed immediately to prevent desiccation‐related artifacts.

### 2.6 Hematological Analysis

Whole blood (~500 μL) was collected from the vena cava into EDTA‐containing tubes (Vacuplast, Brazil). Hematological parameters were analyzed using an automated veterinary hematology analyzer (Bio‐2900 Vet), including red blood cell (RBC) indices, platelet (PLT) counts, hematocrit (HCT), hemoglobin concentration (HGB), and derived parameters such as red cell distribution width (RDW), mean corpuscular volume (MCV), and mean corpuscular hemoglobin concentration (MCHC). A comprehensive list of all abbreviations and their clinical relevance is provided in Table S1.

### 2.7 Biochemical Analyses

Serum biochemical markers were quantified to evaluate systemic homeostatic disturbances and organ‐specific functional impairment. Specifically, transaminases and albumin were measured to assess hepatocellular integrity and hepatic synthetic capacity, while the Evans blue extravasation assay was employed to evaluate intestinal vascular permeability as a proxy for vascular integrity within the mucosal environment.

Serum was obtained by centrifugation of whole blood at 5000 rpm for 10 min at room temperature. Hepatocellular injury and hepatobiliary dysfunction were assessed by measuring alanine aminotransferase (ALT), aspartate aminotransferase (AST), and gamma‐glutamyl transferase (GGT) using commercial kinetic kits. Albumin levels were quantified as an indicator of hepatic synthetic function and systemic homeostasis. Hemoglobin content was evaluated as an indirect marker of tissue hemorrhage or vascular damage. All assays were performed according to the manufacturer's instructions (Bioclin Quibasa, Brazil) with volume adjustments proportional to sample input. Absorbance readings were acquired using a VersaMax microplate reader (Molecular Devices, USA).

Nitric oxide (NO) production was indirectly quantified by measuring nitrite concentrations in tissue homogenate supernatants using the Griess reaction [19]. Absorbance was measured at 540 nm, and nitrite concentrations were calculated based on a sodium nitrite standard curve.

Intestinal vascular permeability was assessed using the Evans blue dye extravasation assay [20]. This assay reflects vascular integrity by quantifying dye extravasation from the bloodstream into the tissue interstitium but does not directly measure epithelial tight junction permeability. Mice received an intravenous injection of 100 μL of a 2% Evans blue solution in PBS. After 1 h, animals were perfused with PBS to remove intravascular dye, and tissues were harvested. Evans blue was extracted with formamide at 60°C for 24 h, and absorbance was measured at 620 nm.

### 2.8 Tissue Processing and Systemic Cytokine Profile

For biochemical and immunological analyses, spleen, the right liver lobe and distal colon were collected, weighed, and homogenized using a TissueLyser LT (Qiagen, Germany). Tissues were processed in an extraction buffer (0.4 M NaCl, 0.05% Tween‐20, 0.5% BSA, 10 mM EDTA, protease inhibitors) at a ratio of 1 mL per 100 mg of tissue. Homogenates were centrifuged at 800 × *g* for 10 min at 4°C, and supernatants were stored at −80°C until analysis. The levels of IL‐2, IL‐4, IL‐6, IL‐10, IL‐17A, IFN‐γ, and TNF were assayed using cytometry bead array (Th1/Th2/Th17 BD Biosciences, USA) according to the manufacturer's instructions. The data were acquired using an LSRFortessa flow cytometer (BD Biosciences, USA), and the results were analyzed in FlowJo software (Tree Star, Ashland, OR). IL‐12 was measured by the sandwich enzyme‐linked immunosorbent (ELISA) kit (R&D Systems, USA) according to the manufacturer's instructions. The absorbance of the samples was determined in a VersaMax ELISA microplate reader (Molecular Devices, USA) at a wavelength of 492 nm, and the cytokine concentration was calculated by interpolation using a standard curve fitted with five parameters of logistic (5‐PL) and expressed in pg/mg of tissue for each sample.

### 2.9 Enzymatic Activity Assays (EPO, MPO, and NAG)

To indirectly quantify the infiltration of specific immune cell subsets into the liver and colon, we measured the activities of eosinophil peroxidase (EPO), myeloperoxidase (MPO), and N‐acetyl‐β‐D‐glucosaminidase (NAG). These enzymatic assays serve as established proxies for eosinophil, neutrophil, and macrophage accumulation, respectively, providing a high‐throughput biochemical assessment of tissue inflammation that complements histological findings. Assays were performed according to previously published protocols [21, 22]. Absorbance was measured using a VersaMax microplate reader, and results were expressed as optical density (OD) values. Results were normalized to tissue weight and expressed as OD per 100 mg of tissue.

### 2.10 Histopathological and Morphometric Analyses

Formalin‐fixed, paraffin‐embedded liver and intestinal tissues were sectioned (5 μm thickness) and stained with hematoxylin and eosin (H&E) for histopathological evaluation. Inflammatory lesions were qualitatively evaluated by a veterinary pathologist, with group allocation masked during analysis. Quantitative morphometric analysis was performed on 20 randomly selected microscopic fields per section at 20× magnification, using Image‐Pro Plus software [23]. Fibrosis was evaluated using Masson's trichrome staining, and collagen deposition was quantified using ImageJ software (version 1.54p). Intestinal mucus production was assessed by Periodic Acid–Schiff (PAS) staining, and mucus‐positive areas were quantified by morphometric analysis.

### 2.11 Tissue Parasite Burden Quantification

DNA was extracted from snap‐frozen liver and intestinal tissues samples (25 ± 2 mg) using a guanidine hydrochloride‐based extraction protocol. Briefly, tissues were lysed in 500 μL guanidine‐HCl lysis buffer (6 M guanidine‐HCl, 50 mM Tris–HCl pH 8.0, 20 mM EDTA) supplemented with proteinase K (200 μg/mL) and incubated at 56°C for 3 h with periodic vortexing. Proteins and polysaccharides were precipitated by adding 150 μL of 7.5 M ammonium acetate and 50 μL of benzyl alcohol, followed by centrifugation at 12000 × *g* for 10 min. DNA was precipitated from the aqueous phase by adding an equal volume of ice‐cold isopropanol, pelleted by centrifugation at 12000 × g for 15 min at 4°C, washed twice with 70% ethanol, air‐dried, and resuspended in 40 μL Tris‐EDTA buffer (10 mM Tris–HCl, 1 mM EDTA, pH 8.0).

DNA concentration and purity were assessed by spectrophotometry (NanoDrop 2000c, Thermo Fisher Scientific, Waltham, MA, USA). Samples were normalized to 50 ng/μL to ensure uniform DNA input across samples. Quantitative PCR was performed using SYBR Green‐based detection in an ABI 7500 Real‐Time PCR System (Applied Biosystems, Foster City, CA, USA). Each 10 μL reaction contained 1× Power SYBR Green PCR Master Mix (Thermo Fisher Scientific), 2 μM of forward primer (5′‐CGAGCTCTTGCCCACACGGGGCT‐3′) and reverse primer (5′‐CCTCCAAGCAGCGGATAGTTTAGG‐3′) targeting a 188 bp fragment of the *T. cruzi* satellite DNA repeat region [24]. Thermal cycling conditions consisted of an initial denaturation at 95°C for 10 min, followed by 40 cycles of 95°C for 15 s and 60°C for 1 min.

Standard curves for absolute quantification were generated using serial 10‐fold dilutions of genomic DNA extracted from 10^7^ *T. cruzi* trypomastigotes. Parasite burden in tissue samples was calculated by interpolation from the standard curve using 7500 Software version 2.0.1 (Thermo Fisher Scientific).

### 2.12 Intestinal Function Assays

To evaluate the functional impact of colonic immunopathology, gastrointestinal motility and fluid handling were assessed. Total intestinal transit time was measured using carmine red as a nonabsorbable marker of propulsive activity, while fecal water content was quantified to identify alterations in intestinal fluid dynamics and absorptive capacity during the course of infection.

Total intestinal transit time was assessed by oral gavage of 100 μL of a 6% carmine red (Sigma‐Aldrich) solution in 0.5% methylcellulose (Sigma‐Aldrich). The time from dye administration to excretion of the first red‐colored fecal pellet was recorded as the total gastrointestinal transit time. To minimize excessive stress, a maximum observation period of 4 h was implemented; animals not excreting red feces within this timeframe were assigned the maximum value. Fecal water content was determined by weighing freshly collected fecal pellets (wet weight), followed by drying at 70°C for 48 h and reweighing to obtain dry weight [25, 26].

### 2.13 Intestinal Microbiota Culture Analysis

To minimize environmental bias in microbiota composition, WT and ST2^−/−^ mice were cohoused in the same animal facility from weaning through the experimental period. Briefly, fecal samples were collected under sterile conditions and serially diluted in PBS 1×. Dilutions were plated on selective and nonselective culture media to quantify cultivable aerobic and anaerobic bacterial groups. Plates were incubated under appropriate atmospheric conditions, and colony‐forming units were expressed as relative proportions of the total cultivable microbiota [27].

### 2.14 Spectral Flow Cytometry

High‐dimensional spectral flow cytometry was utilized to simultaneously characterize the global landscape of splenic adaptive and innate immune cells.

Spleens were removed, placed in ice‐cold sterile PBS, and mechanically disrupted by pressing through 70 μm nylon cell strainers (BD Biosciences, San Jose, CA, USA) into 50 mL tubes. Cell suspensions were centrifuged at 400 × *g* for 6 min at 4°C, supernatants discarded, and pellets resuspended in 4 mL red blood cell lysis buffer (155 mM NH_4_Cl, 10 mM KHCO_3_, 0.1 mM EDTA, pH 7.4) for 5 min at room temperature. Lysis was quenched by adding 10 mL RPMI‐1640 medium (Sigma‐Aldrich), and cells were washed by centrifugation at 400 × *g* for 6 min. Cell pellets were resuspended in complete RPMI medium (supplemented with 10% fetal bovine serum, 100 U/mL penicillin, 100 μg/mL streptomycin, 2 mM L‐glutamine), and viable cell counts were determined using an automated cell counter (Countess II, Life Technologies, Carlsbad, CA, USA) with trypan blue exclusion.

For functional assessment of cytokine‐producing capacity, 1 × 10^6^ splenocytes per sample were incubated in presence of brefeldin A (1:1000 dilution; BD GolgiPlug, BD Biosciences) to block cytokine secretion and enhance intracellular accumulation. Cells were incubated at 37°C in a humidified 5% CO_2_ incubator for 4 h. Cells were washed with PBS and stained with fixable viability dye (1:1000 dilution; BD Horizon Fixable Viability Stain AF700, BD Biosciences) for 10 min at 4°C in the dark. Cells were washed twice with FACS buffer (PBS containing 2% FBS and 2 mM EDTA), then incubated with Fc receptor blocking antibody (anti‐CD16/CD32, clone 93) for 10 min at 4°C to reduce nonspecific antibody binding.

Surface marker staining was performed by incubating cells with fluorochrome‐conjugated antibody cocktails for 20 min at 4°C in the dark. Following surface staining, cells were washed and fixed/permeabilized using the Cytofix/Cytoperm kit (BD Biosciences) according to manufacturer instructions. Following final washes, cells were resuspended in 200 μL PBS and transferred to FACS tubes for acquisition.

The antibody panels included: CD4‐BUV395 (clone GK1.5), CD8‐FITC (clone 53–6.7), CD44‐BV510 (clone IM7), CD11b‐eFluor450 (clone M1/70), Ly6C‐BV570 (clone HK1.4), CD11c‐APC‐Cy7 (clone N418), MHC‐II‐R718 (clone M5/114.15.2), CCR2‐AF647 (clone SA203G11), CX3CR1‐BV650 (clone SA011F11), TNF‐α‐BV421 (clone MP6‐XT22), IFN‐γ‐PE‐CF594 (clone XMG1.2), IL‐4‐BV711 (clone 11B11), CD45‐PE‐Cy7 (clone 30‐F11), IL‐10‐APC (clone JES5‐16E3), IL‐12‐PE (clone C17.8), F4/80‐PERCP (clone BM8), IL‐17A‐BV650 (clone TC11‐18H10.1). All antibodies were obtained from BD Biosciences or BioLegend (San Diego, CA, USA). For each sample, 100 000 events were acquired on a BD FACSymphony A5 flow cytometer (BD Biosciences). Flow cytometry data were analyzed using FlowJo software version 10.8.1 (BD Biosciences). Spectral unmixing was performed using a reference library built from single‐stained controls (UltraComp eBeads) and a live‐cell autofluorescence reference. Gating strategies shown in Figures S4 and S10, with expanded legends describing the rationale for each step. Briefly, for T‐cell immunophenotyping, cells were sequentially gated for lymphocytes (FSC‐A vs. SSC‐A), singlets (SSC‐H vs. SSC‐A), and viable cells (Viability Stain). Within the CD45+ population, CD4+ and CD8+ T‐cell subsets were identified. For myeloid profiling, CD11b + cells within the live CD45+ population were subdivided: patrolling monocytes (Ly6C^low^ CX3CR1+ CD11b+), classical monocytes (Ly6C^high^ CCR2+ CD11b+), DCs (CD11c + MHC‐II+ CD11b+), and splenic macrophages (F4/80+ Cd11b+).

### 2.15 Statistical and Computational Analysis

Statistical analyses and data visualization were performed using R software (version 4.4.3) with the tidyverse and ggplot2 packages [28]. Data distribution was assessed using the Shapiro–Wilk test. For normally distributed data, comparisons between two groups were performed using an unpaired Student's *t*‐test, whereas multiple‐group comparisons were conducted via one‐way or two‐way ANOVA, followed by Tukey's posthoc test. Nonnormally distributed data were analyzed using the Mann–Whitney *U* test (two groups) or the Kruskal–Wallis test (multiple groups). Results are expressed as mean ± SEM, and *p* ≤ 0.05 was considered statistically significant.

High‐dimensional flow cytometry data were analyzed using the cytofkit2 R package, employing t‐distributed stochastic neighbor embedding (t‐SNE) for dimensionality reduction and PhenoGraph for unsupervised clustering [29].

## 3 Results

### 3.1 The IL‐33/ST2 Axis Modulates Clinical Progression and Hematological Homeostasis During *T. cruzi* Infection

**FIGURE 1:**
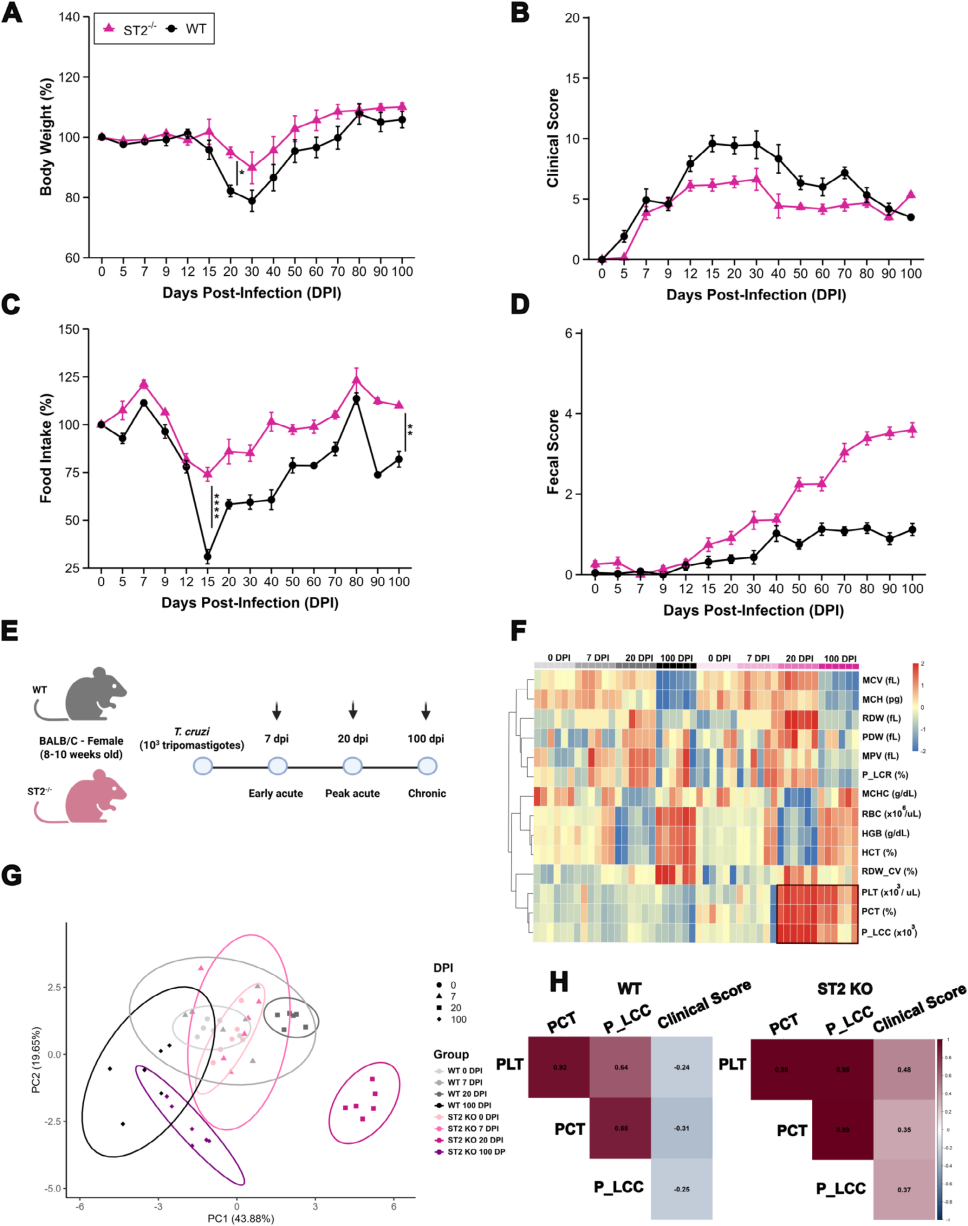
Clinical and hematological dynamics during *T. cruzi* infection in WT and ST2^−/−^ mice. Animals were infected with 1000 *T. cruzi* blood trypomastigotes and monitored for up to 100 days. (A) Percentage change in body weight. (B) Clinical score. (C) Percentage change in food intake (data pooled from two cages; *n* = 3 mice per cage). (D) Fecal score. (E) Experimental schematic highlighting infection timeline and key analytical time points (7, 20, and 100 dpi). (F) Heatmap displaying hematological parameters; the box highlights the platelet compartment expansion in ST2^−/−^ mice. A detailed description of hematological parameters and their clinical relevance is provided in Table S1. (G) Principal component analysis (PCA) of hematological profiles across genotypes and infection stages. Symbols indicate the kinetics of infection (Circles: 0 DPI; Triangles: 7 DPI; Squares: 20 DPI; Diamonds: 100 DPI), while Colors distinguish the experimental groups and their baseline (Gray shades: Wild‐type trajectory; Pink to Purple shades: ST2‐deficient trajectory). Ellipses represent 95% confidence intervals for each group. (H) Spearman correlation matrices depicting associations between platelet‐related parameters (PLT, PCT, P‐LCC) and clinical scores in WT (left) and ST2^−/−^ (right) mice. Data represent mean ± SEM from *N* = 6 biological replicates per group, representative of two independent experiments. *p < 0.05, **p < 0.01, ****p < 0.0001 (Two‐way ANOVA with Tukey's post hoc test). ST2^−/−^, ST2‐deficient; WT, wild‐type.

To investigate the role of the IL‐33/ST2 pathway in the clinical course of experimental *T. cruzi* infection, we monitored ST2‐deficient (ST2^−/−^) and wild‐type (WT) BALB/c mice for up to 100 days postinfection (dpi). Both genotypes developed comparable overall trajectories of disease severity, with the onset of clinical manifestations occurring between 15 and 30 dpi, characterized by transient weight loss and increased clinical scores (Figure 1A,B). ST2^−/−^ mice maintained food intake throughout infection (Figure 1C) despite comparable body weight loss, which may reflect distinct metabolic or behavioral patterns specific to ST2 deficiency (Figure 1C). This occurred despite similar body weight trajectories between genotypes, indicating distinct physiological or behavioral adaptations in the absence of ST2 signaling. During chronic infection, ST2^−/−^ mice developed elevated fecal scores characterized by mucoid stool consistency (Figure 1D), consistent with altered intestinal function during the chronic phase.

Given these genotype‐specific clinical features, analyses were subsequently focused on three predefined time points: early acute (7 dpi), peak acute (20 dpi), and chronic infection (100 dpi) [14] (Figure 1E–G). Hematological profiling revealed that both WT and ST2^−/−^ mice developed systemic hematological alterations at peak infection (20 dpi), characterized by increased red cell distribution width (RDW) and mean corpuscular volume (MCV), consistent with anisocytosis and macrocytic remodeling. During the chronic phase, elevations in red blood cell count (RBC), hematocrit (HCT), hemoglobin concentration (HGB), and mean corpuscular hemoglobin concentration (MCHC) were observed in both genotypes (Figure 1F). This pattern is compatible with a compensatory erythropoietic response (Table S1).

A marked divergence emerged within the platelet compartment during the transition to chronic infection. ST2^−/−^ mice uniquely exhibited sustained and coordinated expansion of platelet‐related parameters, including platelet count (PLT), plateletcrit (PCT), and large platelet count (P‐LCC) (Figure 1F, highlighted box). Principal component analysis (PCA) further resolved this divergence, with ST2^−/−^ mice clustering distinctly from both their baseline (0 dpi) and WT counterparts during chronic infection (Figure 1G). Importantly, platelet expansion correlated positively with clinical severity exclusively in ST2^−/−^ mice, whereas no such association was observed in WT animals (Figure 1H). Together with the hepatosplenomegaly noted in ST2^−/−^ mice (Figure S1), these findings point to a disruption of systemic homeostasis in the absence of ST2 signaling.

### 3.2 IL‐33/ST2 Signaling Preserves Hepatic Function and Restrains Chronic Inflammation During *T. cruzi* Infection

**FIGURE 2:**
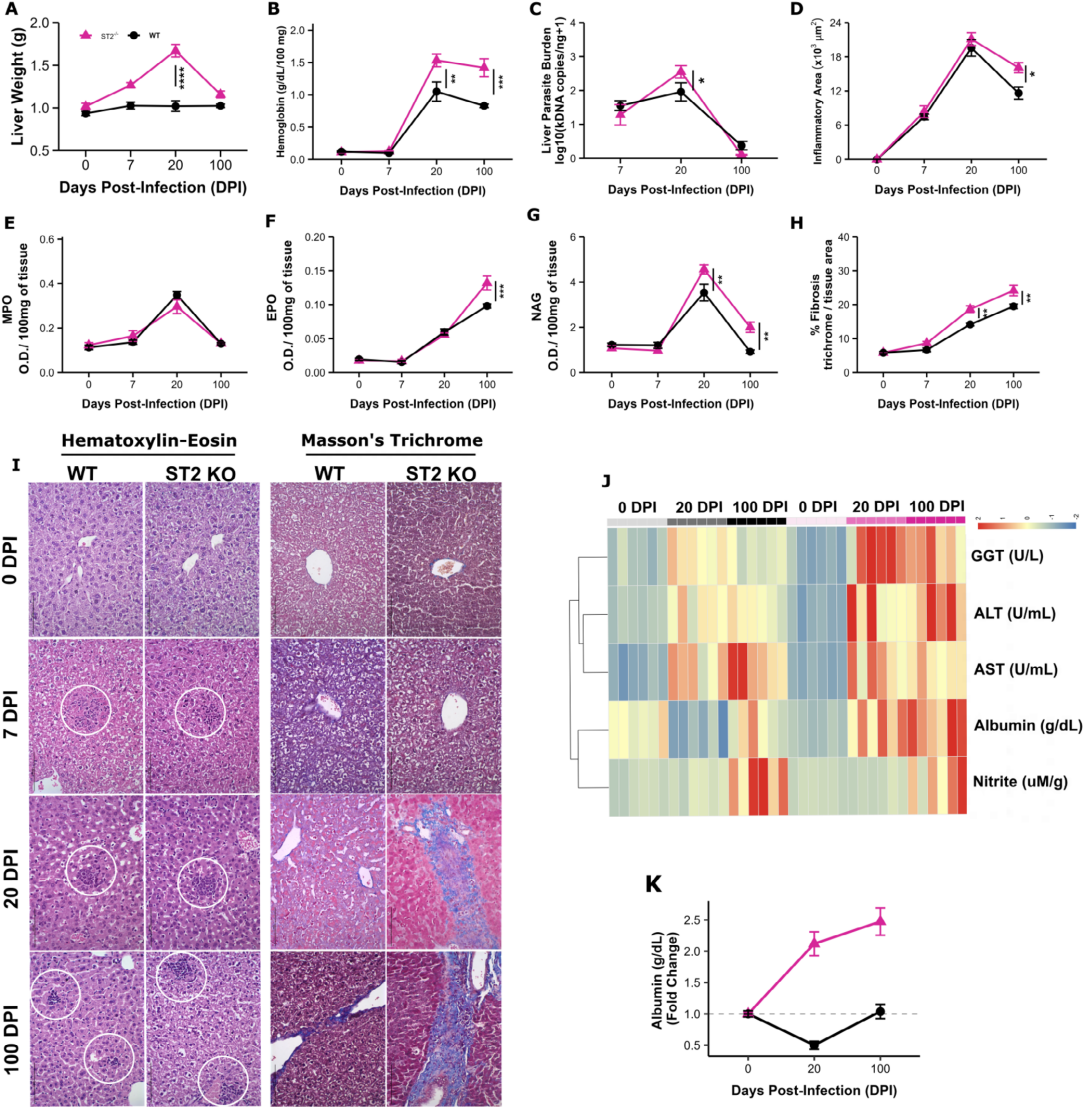
ST2 deficiency exacerbates liver inflammation, fibrosis, and functional impairment during *T. cruzi* infection. WT and ST2^−/−^ mice were analyzed over 100 days postinfection. (A) Total liver weight (g). (B) Hemoglobin concentration in tissue homogenates. (C) Hepatic parasite burden quantified by kDNA copies. (D) Morphometric quantification of the total inflammatory area. (E–G) Activity of Myeloperoxidase (MPO), Eosinophil Peroxidase (EPO), and N‐acetylglucosaminidase (NAG) in hepatic tissue. (H) Percentage of collagen deposition (fibrotic area). (I) Representative histological sections of liver tissue stained with Hematoxylin & Eosin (H&E) and Masson's Trichrome. White circles highlight multifocal inflammatory aggregates; blue staining indicates collagen fibers. Scale bar: 100 μm. (J) Heatmap of systemic biochemical markers: Aspartate aminotransferase (AST, marker of liver or muscle damage), alanine aminotransferase (ALT, hepatocellular injury marker), nitrite levels (indirect marker of nitric oxide production), gamma‐glutamyl transferase (GGT, marker of cholestatic injury and oxidative stress), and albumin (marker of hepatic synthetic function and acute‐phase response). (K) Fold change in serum Albumin levels relative to baseline. Data represent mean ± SEM from *N* = 5–6 biological replicates per group. **p < 0.01, ***p < 0.001, ****p < 0.0001 (Two‐way ANOVA with Tukey's post hoc test). O.D., optical density; ST2^−/−^, ST2‐deficient; WT, wild‐type.

The systemic and hematological alterations observed in ST2‐deficient mice prompted a focused evaluation of hepatic structure and function, given the central role of the liver in hematopoiesis, metabolism, and vascular regulation. The liver serves as a critical immunological and metabolic organ during *T. cruzi* infection, balancing antimicrobial defense with the maintenance of systemic homeostasis [30]. Given the systemic hematological disturbances and hepatomegaly observed in ST2^−/−^ mice (Figure 2A), we investigated whether hepatic alterations paralleled this systemic phenotype.

Hepatic hemoglobin content was elevated in ST2^−/−^ mice throughout chronic infection (Figure 2B), consistent with persistent vascular leakage or congestion within the liver parenchyma. This vascular alteration aligns with the platelet abnormalities observed systemically.

Increased hepatic parasite burden was detected at peak infection (20 dpi) in ST2^−/−^ mice compared with WT counterparts (Figure 2C). Morphometric analysis further demonstrated a progressive expansion of inflammatory areas in the livers of ST2^−/−^ mice (Figure 2D). Notably, inflammatory infiltrates were associated with increased eosinophil peroxidase (EPO) and N‐acetylglucosaminidase (NAG) activities, whereas myeloperoxidase (MPO) activity remained comparable between genotypes (Figure 2E–G). These data indicate that ST2 deficiency does not alter neutrophil‐associated activity but results in elevated enzymatic markers typically associated with eosinophils and macrophages within the hepatic microenvironment. Concomitantly, ST2^−/−^ mice exhibited accelerated fibrotic remodeling and increased collagen deposition (Figure 2H,I).

Histopathological analysis further revealed heterogeneous, large inflammatory foci distributed throughout both the parenchyma and perivascular regions (Figure 2I; Figure S2). By 100 dpi, ST2^−/−^ mice developed significantly enlarged inflammatory aggregates dominated by lymphocytic infiltrates, which persisted across the hepatic parenchyma. This shift from mixed inflammatory infiltrates during acute infection to lymphocyte‐predominant lesions during chronic stages is consistent with sustained immune‐mediated hepatic inflammation, rather than resolution of infection.

To determine whether these structural alterations were accompanied by functional hepatic impairment, a comprehensive biochemical analysis was performed (Figure 2J). While aspartate aminotransferase (AST) levels remained comparable across groups, ST2^−/−^ mice displayed significantly elevated alanine aminotransferase (ALT) levels, indicating ongoing hepatocellular injury. Nitrite levels exhibited tighter regulation in WT mice, whereas ST2^−/−^ mice showed greater variability, consistent with less coordinated nitric oxide–associated responses. In addition, ST2^−/−^ mice exhibited sustained elevations in gamma‐glutamyl transferase (GGT) during the chronic phase (Figure 2J), a marker commonly associated with biliary stress or cholestatic injury.

Serum albumin levels displayed distinct temporal dynamics between genotypes. WT mice showed a transient reduction during acute infection followed by normalization during chronic stages, consistent with a classical acute‐phase response. In contrast, ST2^−/−^ mice exhibited a progressive and sustained increase in serum albumin levels throughout chronic infection (Figure 2K). This persistent elevation aligns with altered hepatic synthetic dynamics. While the precise nature of this response remains to be fully elucidated, it coincides with sustained vascular dysfunction rather than a return to baseline homeostasis.

### 3.3 IL‐33/ST2 Signaling Regulates Colonic Inflammation, Structural Remodeling, and Intestinal Function During *T. cruzi* Infection

The hepatic structural and biochemical alterations described above indicate that loss of ST2 signaling compromises hepatic homeostatic control and extends beyond the liver, pointing to dysfunction along the hepato–intestinal axis. Considering the marked hepatic dysfunction observed in ST2‐deficient mice, characterized by altered biochemical markers associated with hepato–intestinal homeostasis, we next investigated whether impairment of IL‐33/ST2 signaling extended to the colonic compartment. In both experimental models and human Chagas disease, the colon represents a major target organ, where chronic inflammation, neuromuscular dysfunction, and long‐term tissue remodeling contribute to gastrointestinal morbidity [31, 32].

**FIGURE 3:**
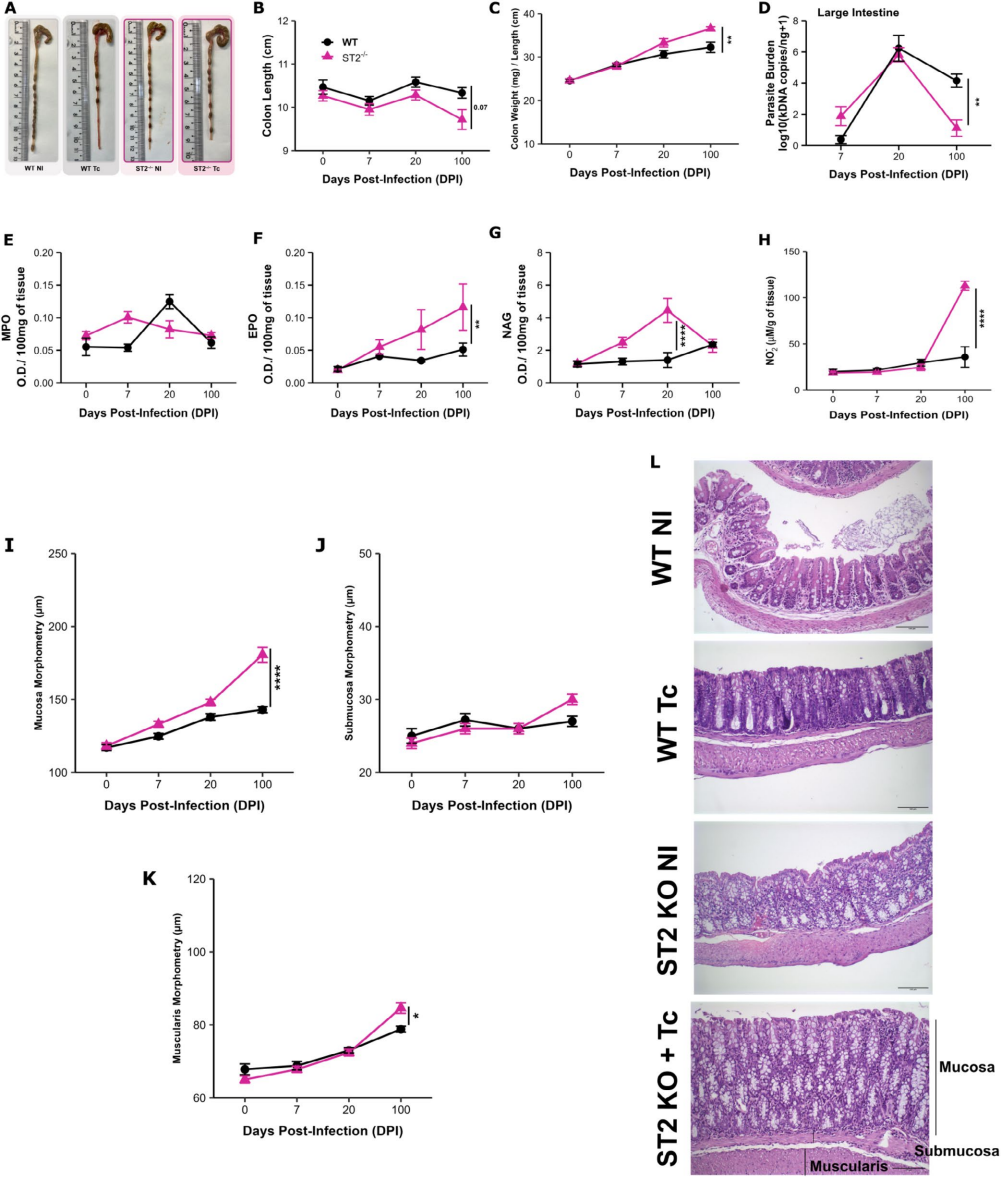
IL‐33/ST2 signaling modulates colonic parasitism and inflammation during *T. cruzi* infection. (A) Representative macroscopic colon images. (B) Colon length (cm). (C) Colon weight/length ratio as a marker of tissue remodeling. (D) Colonic parasite burden (kDNA copies). (E–G) Activity of MPO (neutrophils), EPO (eosinophils), and NAG (macrophages). (H) Nitric oxide (NO) production in colonic tissue. (I–K) Morphometric analysis of mucosal, submucosal, and muscularis thickness. (L) Representative H&E sections highlighting intestinal layer distribution. Note the expansion of the layers in ST2^−/−^ infected mice. Scale bar: 100 μm. Data represent mean ± SEM from *N* = 5–6 biological replicates/group. **p < 0.01, ****p < 0.0001 (Two‐way ANOVA with Tukey's post hoc). O.D., optical density; ST2^−/−^, ST2‐deficient; WT, wild‐type.

Macroscopic and morphometric analyses revealed progressive colonic alterations in ST2^−/−^ mice. Although colon length showed only a modest tendency toward shortening during infection (Figure 3A,B), ST2‐deficient mice exhibited a significantly increased colon weight‐to‐length ratio during the chronic phase (Figure 3C), suggestive of muscularis hypertrophy and/or inflammatory edema.

In contrast to the liver, the colonic parasite burden was significantly reduced in ST2^−/−^ mice during chronic infection (Figure 3D), highlighting a tissue‐specific decoupling of pathogen clearance and immunopathology.

Analysis of the inflammatory profile revealed genotype‐specific differences within the colonic compartment. Neutrophil‐associated activity, assessed by myeloperoxidase (MPO), remained comparable between infected WT and ST2^−/−^ mice (Figure 3E). In contrast, ST2^−/−^ mice exhibited increased EPO activity during the chronic phase (Figure 3F), as well as a transient increase in NAG activity during acute infection (Figure 3G). These enzymatic profiles suggest a distinct inflammatory milieu in the ST2‐deficient colonic compartment. Notably, ST2^−/−^ mice produced markedly elevated levels of nitric oxide (NO) in colonic tissue (Figure 3H), a finding consistent with the observed reduction in parasite burden. This potent inflammatory environment coincided with increased mucosal and muscularis thickness in ST2‐deficient mice during the chronic phase (Figure 3I–L). These data suggest that while ST2 deficiency is associated with enhanced parasiticidal activity, potentially mediated by NO, it does not prevent the development of progressive colonic immunopathology.

**FIGURE 4:**
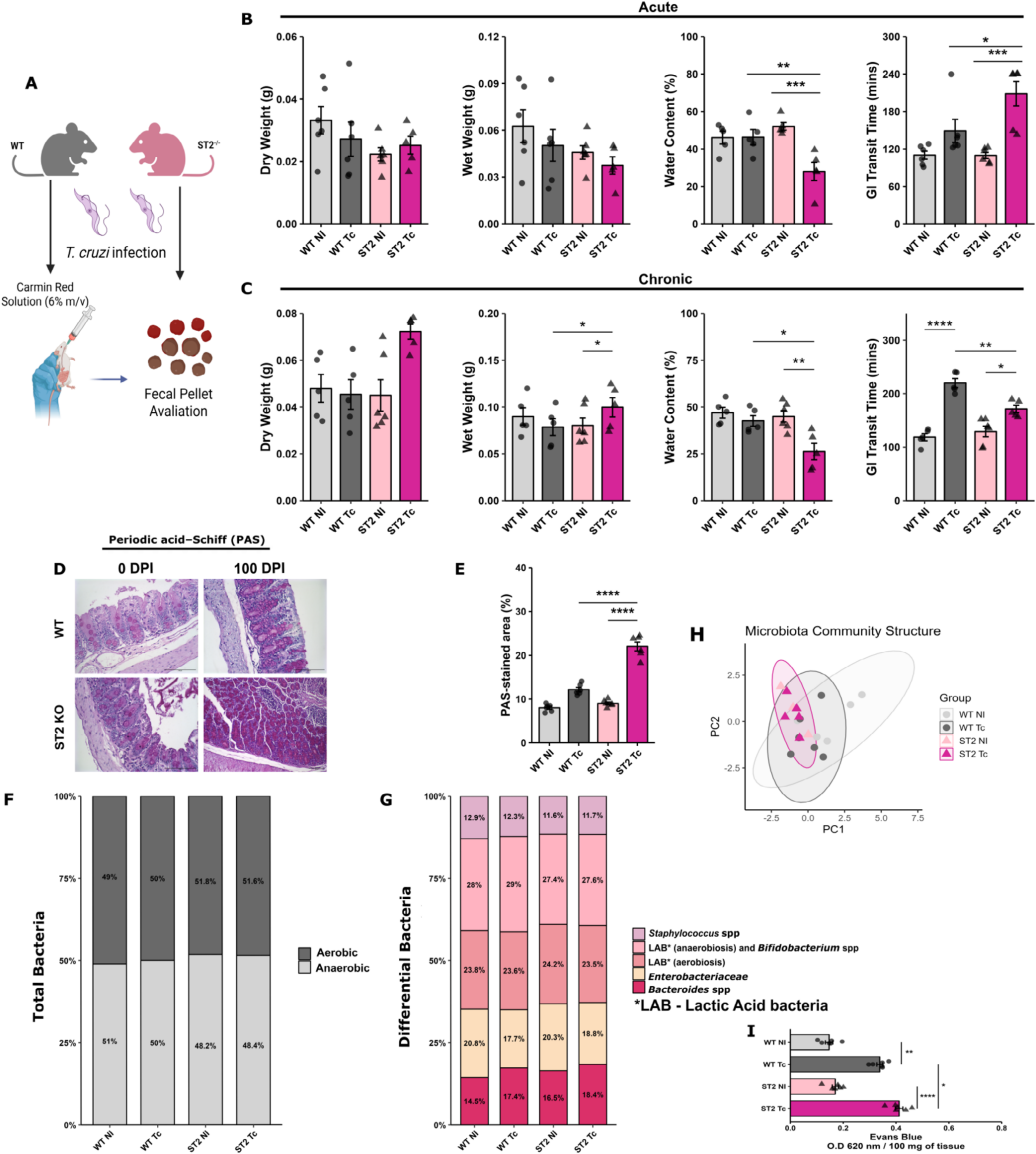
ST2 deficiency disrupts intestinal function and homeostasis during *T. cruzi* infection. (A) Experimental schematic of the carmine red transit assay. (B, C) Functional analysis (dry/wet weight, water content, and transit time) during acute and chronic phases. (D, E) PAS‐stained sections and quantification of goblet cells/mucus production by morphometric analysis. PAS positivity reflects increased mucosal thickness and total mucus‐containing epithelial area. (F) Total aerobic and anaerobic bacterial loads cultured on blood agar. (G) Differential bacterial populations cultured on selective media: Mannitol agar (*Staphylococcus* spp.), MacConkey agar (Enterobacteriaceae), esculin bile agar (*Streptococcus* spp. and *Enterococcus* spp.), De Man, Rogosa and Sharpe (MRS) agar in aerobic conditions (aerobic lactic acid bacteria), and MRS agar in anaerobic conditions (anaerobic lactic acid bacteria including *Bifidobacterium* spp.). (H) Principal component analysis (PCA) of cultivable microbiota profiles showing no genotype‐specific clustering. (I) Evans blue accumulation as an indirect indicator of tissue vascular leakage. Scale bar: 100 μm. Data represent mean ± SEM from *N* = 5 to 6 biological replicates/group. *p < 0.05, **p < 0.01, ***p < 0.001, ****p < 0.0001. Figure (A–D): Two‐way ANOVA with Tukey's post hoc. Figure (F): One‐way ANOVA with Tukey's post hoc. ST2^−/−^, ST2‐deficient; WT, wild‐type.

To determine whether these structural changes were associated with functional impairment, gastrointestinal transit and fecal water homeostasis were assessed (Figure 4A–C). During the acute phase, ST2^−/−^ mice exhibited a reduction in fecal water content despite comparable stool mass, occurring alongside alterations in colonic motility (Figure 4B). This phenotype suggests a transient alteration in intestinal fluid dynamics during the early stages of infection. However, this pattern was not sustained into the chronic phase, where ST2^−/−^ mice developed mucoid stools with increased wet weight (Figure 4C), consistent with the clinical fecal scores and the progression of colonic inflammation (Figure 1D).

To assess epithelial alterations, we performed Periodic Acid–Schiff (PAS) staining. At 100 dpi, ST2^−/−^ mice exhibited an expansion of PAS‐positive areas (Figure 4D,E), which aligned with the overall mucosal hypertrophy and remodeling observed under sustained inflammation.

Despite these marked structural and functional changes, analysis of cultivable fecal microbiota revealed no significant differences in aerobic or anaerobic bacterial proportions, nor in overall microbial community structure as assessed by principal component analysis, between WT and ST2^−/−^ mice (Figure 4F–H). In contrast, intestinal vascular permeability was significantly increased in ST2^−/−^ mice, as demonstrated by increased Evans blue extravasation (Figure 4I), a measure of vascular permeability.

The concurrence of increased intestinal vascular permeability and systemic vascular alterations, including platelet expansion and hyperalbuminemia, highlights a multiorgan involvement in ST2‐deficient mice. These intestinal abnormalities occurred in the absence of any significant alterations in the cultivable aerobic and anaerobic bacterial compartments, suggesting that the observed intestinal disturbances are more closely associated with immune dysregulation than with alterations in the cultivable microbiota.

### 3.4 The IL‐33/ST2 Axis Orchestrates a Balanced T Cell Landscape and Prevents Chronic Th1 Polarization During *T. cruzi* Infection

To evaluate how the absence of ST2 signaling impacts the systemic inflammatory environment over time, we first quantified a panel of key immunoregulatory and effector cytokines in the spleen using cytometric bead array (CBA) and sandwich ELISA (Figure S3). During the early acute phase (7 dpi), ST2‐deficient mice demonstrated an early acceleration of type 1 responses, characterized by significantly higher levels of systemic IFN‐γ compared to WT counterparts. By the peak of the acute phase (20 dpi), this inflammatory skewing was consolidated; ST2^−/−^ mice exhibited marked elevations in pro‐inflammatory signals, including IFN‐γ, TNF, IL‐12, and IL‐6. Concomitantly, while WT mice mounted a counter‐regulatory response at 20 dpi with significant upregulations of IL‐10 and IL‐17, ST2‐deficient mice showed a substantial impairment in these regulatory and tissue‐protective niches, demonstrating a failure to sustain stable counter‐regulatory networks. This unbalanced profile in ST2^−/−^ mice persisted into the chronic stage (100 dpi), sustained by elevated IFN‐γ and TNF‐ along with lowered IL‐10 availability (Figure S3).

**FIGURE 5:**
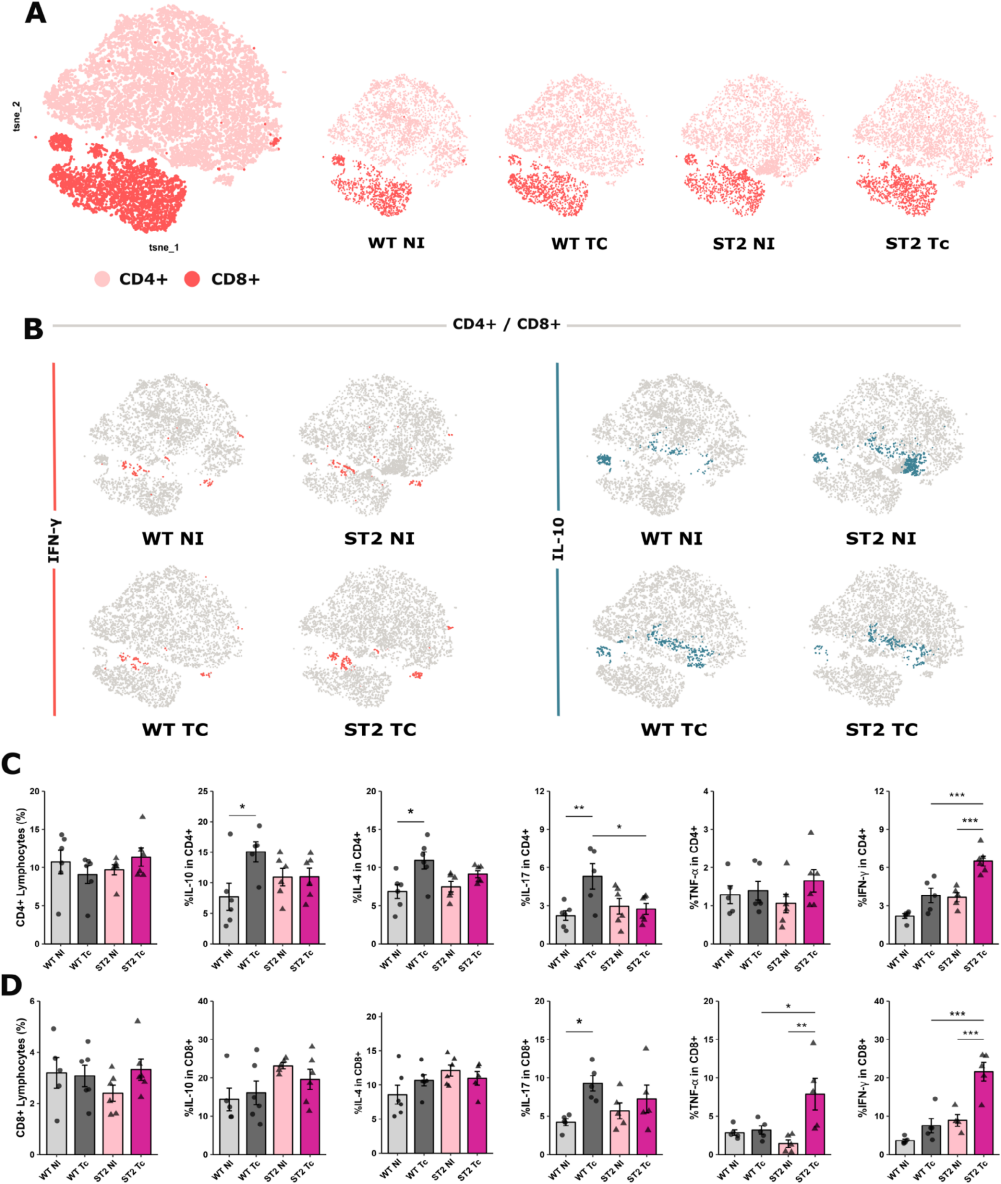
IL‐33/ST2 signaling modulates T‐cell immune landscapes during acute (7 dpi) *T. cruzi* infection. (A) Global t‐SNE analysis showed populations of CD4+ T lymphocytes (light pink) and CD8+ T lymphocytes (red). To ensure balanced representation, equal numbers of events from all experimental groups were concatenated prior to dimensionality reduction. (B) Cytokine expression projected onto the t‐SNE manifold highlighting IL‐10 (blue) and IFN‐γ (red)–expressing clusters. (C) Frequency and cytokine profile of CD4+ T cells expressing IL‐4, IL‐10, IL‐17, TNF, and IFN‐γ. (D) Frequency and cytokine profile of CD8+ T cells expressing IL‐4, IL‐10, IL‐17, TNF, and IFN‐γ. Data represent mean ± SEM from *N* = 5 to 6 biological replicates/group. **p < 0.05, **p < 0.01, ***p < 0.001, ****p < 0.0001 (One‐way ANOVA with Tukey's post hoc). ST2^−/−^, ST2‐deficient; WT, wild‐type.

**FIGURE 6:**
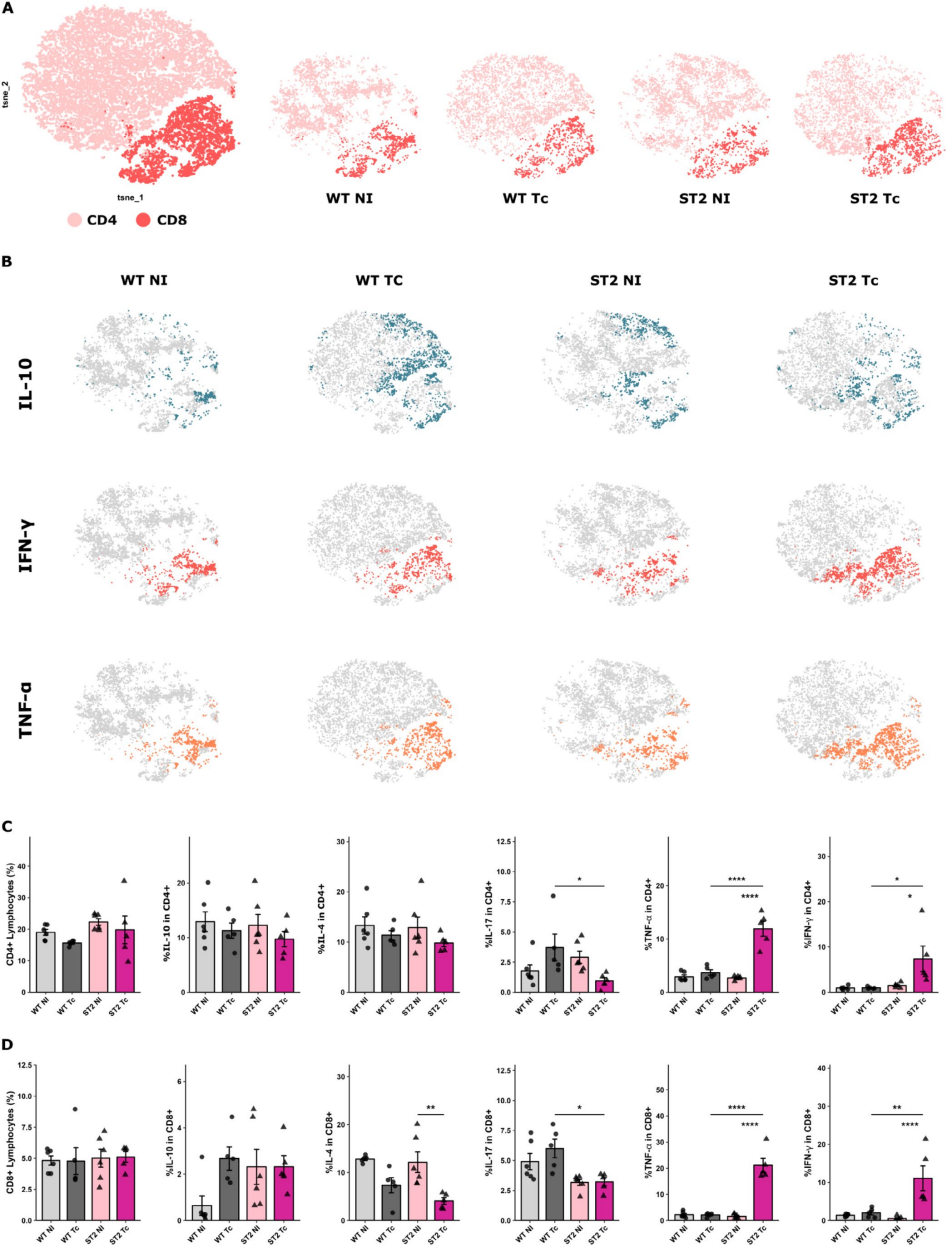
ST2 deficiency enforces chronic (100 dpi) inflammatory T‐cell reprogramming during *T. cruzi* infection. (A) Global t‐SNE analysis showed populations of CD4+ T lymphocytes (light pink) and CD8+ T lymphocytes (red). To ensure balanced representation, equal numbers of events from all experimental groups were concatenated prior to dimensionality reduction. (B) Cytokine expression projected onto the t‐SNE manifold highlighting IL‐10 (blue), TNF (orange) and IFN‐γ (red)–expressing clusters. (C) Frequency and cytokine profile of CD4+ T cells expressing IL‐4, IL‐10, IL‐17, TNF, and IFN‐γ. (D) Frequency and cytokine profile of CD8+ T cells expressing IL‐4, IL‐10, IL‐17, TNF, and IFN‐γ. Data represent mean ± SEM from *N* = 5 to 6 biological replicates/group. *p < 0.05, **p < 0.01, ***p < 0.001, ****p < 0.0001 (One‐way ANOVA with Tukey's post hoc). ST2^−/−^, ST2‐deficient; WT, wild‐type.

To identify the cellular configurations associated with this altered systemic milieu, we utilized high‐dimensional spectral flow cytometry to characterize splenic CD4^+^ and CD8^+^ T cells during the acute and chronic phases of infection (Figures 5 and 6). Concatenated t‐SNE analysis revealed a conserved global topology of CD4^+^ and CD8^+^ T cell populations across experimental groups, indicating that ST2 deficiency does not alter gross T cell recruitment or lineage distribution during acute infection (Figure 5A). However, projection of cytokine expression onto the t‐SNE manifold uncovered distinct functional activation states between genotypes. In WT mice, a discrete CD4^+^ T cell cluster exhibiting an elevated IL‐10 signal emerged during acute infection, occupying a defined marginal region of the embedding (Figure 5B). In contrast, this IL‐10–signal was less prominent in ST2^−/−^ mice, corroborating the systemic deficit observed in our protein assays. Conversely, IFN‐γ–expressing T cells displayed a broader spatial distribution and higher density in ST2‐deficient mice, consistent with the early polarization toward a pro‐inflammatory state.

Quantitative analysis confirmed that WT mice mounted a balanced, polyfunctional CD4^+^ response characterized by the coordinated upregulation of IL‐10, IL‐4, and IL‐17 (Figure 5C). In contrast, ST2^−/−^ CD4^+^ T cells failed to engage these circuits and instead showed a selective increase in IFN‐γ expression, reinforcing the early shift toward a Th1‐type profile. Cytokine ratio analyses further confirmed Th1 dominance over regulatory and Th2/Th17 cytokines in ST2^−/−^ mice (Figures S5 and S6). Examination of the CD8^+^ T cell compartment revealed a parallel divergence (Figure 5D); whereas WT mice increased expression of IL‐10 and IL‐17 during acute infection, ST2^−/−^ mice exhibited robust TNF and IFN‐γ production, consistent with enhanced cytotoxic potential in the absence of ST2 signaling (Figure S7).

During the chronic phase of infection (100 dpi), t‐SNE analysis demonstrated increased heterogeneity and further divergence of T cell profiles between genotypes (Figure 6A). Cytokine projection highlighted the expansion of TNF‐ and IFN‐γ‐producing clusters, particularly in ST2^−/−^ mice (Figure 6B). Notably, the early absence of IL‐10–enriched niches was associated with a persistent deficiency of IL‐10–producing T cells in chronically infected ST2^−/−^ mice, whereas WT mice maintained distinct regulatory niches. Consistent with these patterns, CD4^+^ T cells from ST2^−/−^ mice showed a marked reduction in IL‐17 expression, accompanied by sustained elevations of TNF and IFN‐γ levels (Figure 6C). CD8^+^ T cells similarly lacked robust IL‐4 or IL‐17 induction while maintaining a dominant inflammatory cytotoxic signature (Figure 6D), supported by sustained Th1 dominance over Th2 and Th17 responses in both compartments (Figures S8 and S9). Collectively, these findings suggest that while lymphoid recruitment occurs independently of ST2, its absence alters the early functional organization of adaptive immune responses, impeding regulatory counterbalance and permitting sustained Th1 and cytotoxic polarization.

### 3.5 IL‐33/ST2 Signaling Orchestrates Myeloid Functional Polarization to Preserve Vascular and Tissue Homeostasis During *T. cruzi* Infection

To identify the upstream immune alterations associated with the sustained inflammatory T‐cell polarization observed in ST2‐deficient mice, we performed a high‐dimensional characterization of the splenic myeloid compartment. Myeloid cells, particularly monocytes (MOs) and dendritic cells (DCs), function as central regulators of adaptive immunity through pathogen sensing, antigen presentation, and cytokine production, including IL‐12.

**FIGURE 7:**
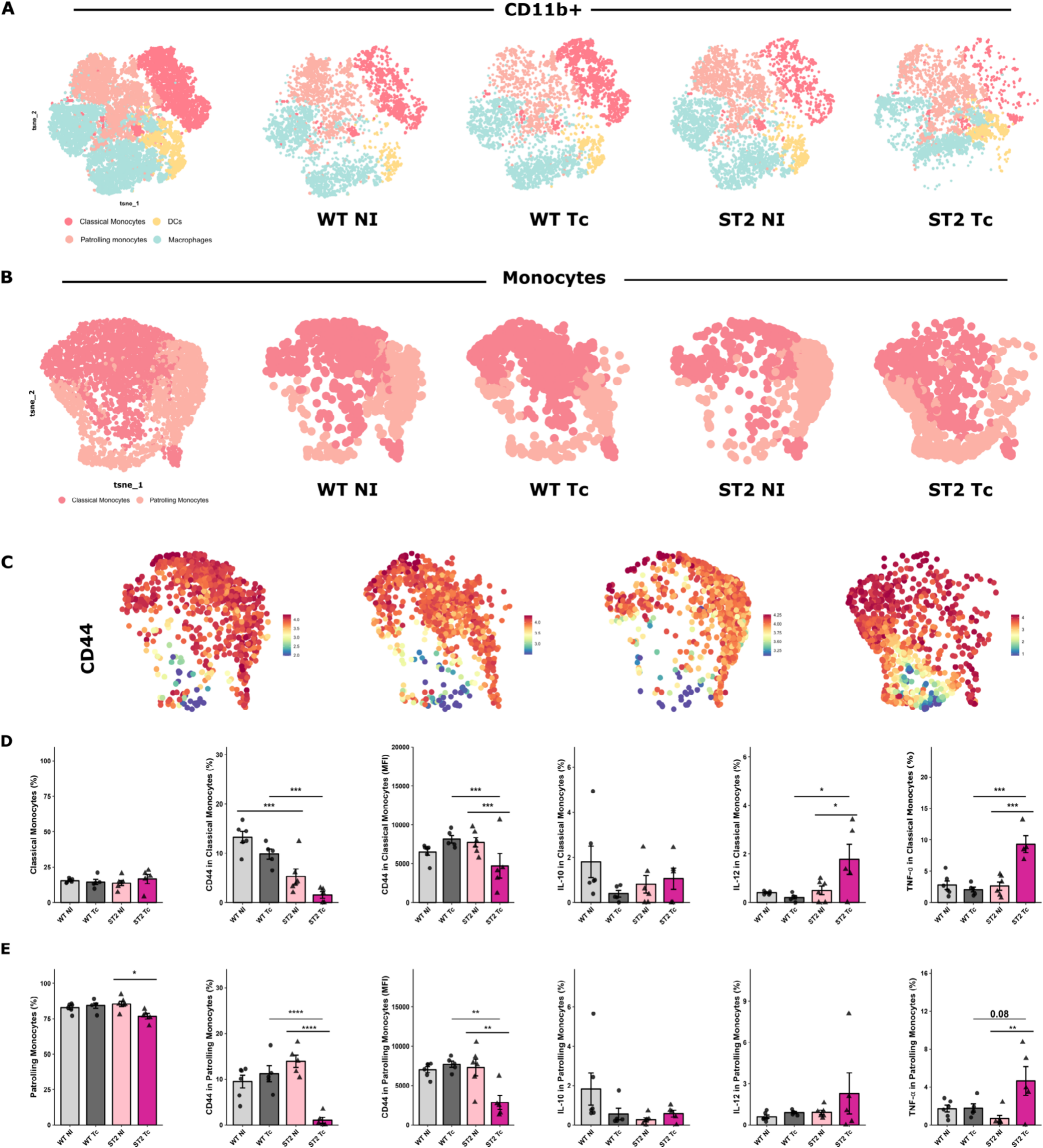
IL‐33/ST2 signaling preserves monocyte organization and homeostatic programs during *T. cruzi* infection. (A) t‐SNE analysis of splenic CD11b^+^ myeloid cells showing macrophages, dendritic cells, classical monocytes, and patrolling monocytes across experimental groups. (B) t‐SNE projection of monocyte subsets highlighting classical and patrolling monocytes. (C) CD44 expression overlaid on monocyte t‐SNE embeddings. (D, E) Frequency and cytokine expression profiles of classical (D) and patrolling (E) monocytes. Data represent mean ± SEM from *N* = 5 to 6 mice/group. *p < 0.05, **p < 0.01, ***p < 0.001 (One‐way ANOVA with Tukey's post hoc). ST2^−/−^, ST2‐deficient; WT, wild‐type.

Dimensionality‐reduction analysis of splenic CD11b^+^ cells identified splenic macrophages (SMs), classical (inflammatory—Ly6C^high^) and nonclassical (patrolling—Ly6C^low^) monocytes, and dendritic cells (DCs) across all experimental groups (Figure 7A). In WT mice, both naïve and infected, the global myeloid landscape remained largely conserved, indicating preservation of innate immune architecture despite infection. In contrast, ST2^−/−^ infected mice displayed a marked alteration in myeloid cluster density and spatial distribution, consistent with disruption of normal myeloid organization.

Feature overlay analysis revealed that CD44 expression as a major organizing principle of monocyte heterogeneity, with graded CD44 expression delineating functional states within both monocyte subsets (Figure 7B,C). CD44, a hyaluronic acid receptor, is involved in monocyte adhesion, migration, endothelial interaction, and differentiation into macrophages or DCs [33]. In WT mice, both monocyte subsets aligned along a coherent CD44 gradient, consistent with coordinated maturation and functional diversification. In ST2^−/−^ infected mice, this CD44‐associated organization was markedly reduced, indicating altered regulation of adhesive and migratory programs during infection.

Quantitative subset analysis revealed that ST2^−/−^ mice exhibited a selective reduction in patrolling monocyte (Ly6C^low^) frequency during infection, while classical monocyte (Ly6C^high^) frequencies remained comparable to WT counterparts (Figure 7D,E). Patrolling monocytes are associated with endothelial surveillance and vascular homeostasis [34, 35]. Our results show that ST2 deficiency is associated with a selective reduction of the patrolling monocyte subset, which canonically mediates vascular surveillance, potentially consistent with reduced endothelial surveillance capacity. In parallel, both monocyte subsets from infected ST2^−/−^ mice exhibited increased expression of IL‐12 and TNF relative to naive or WT counterparts (Figure 7D,E), consistent with a shift toward a pro‐inflammatory functional state.

**FIGURE 8:**
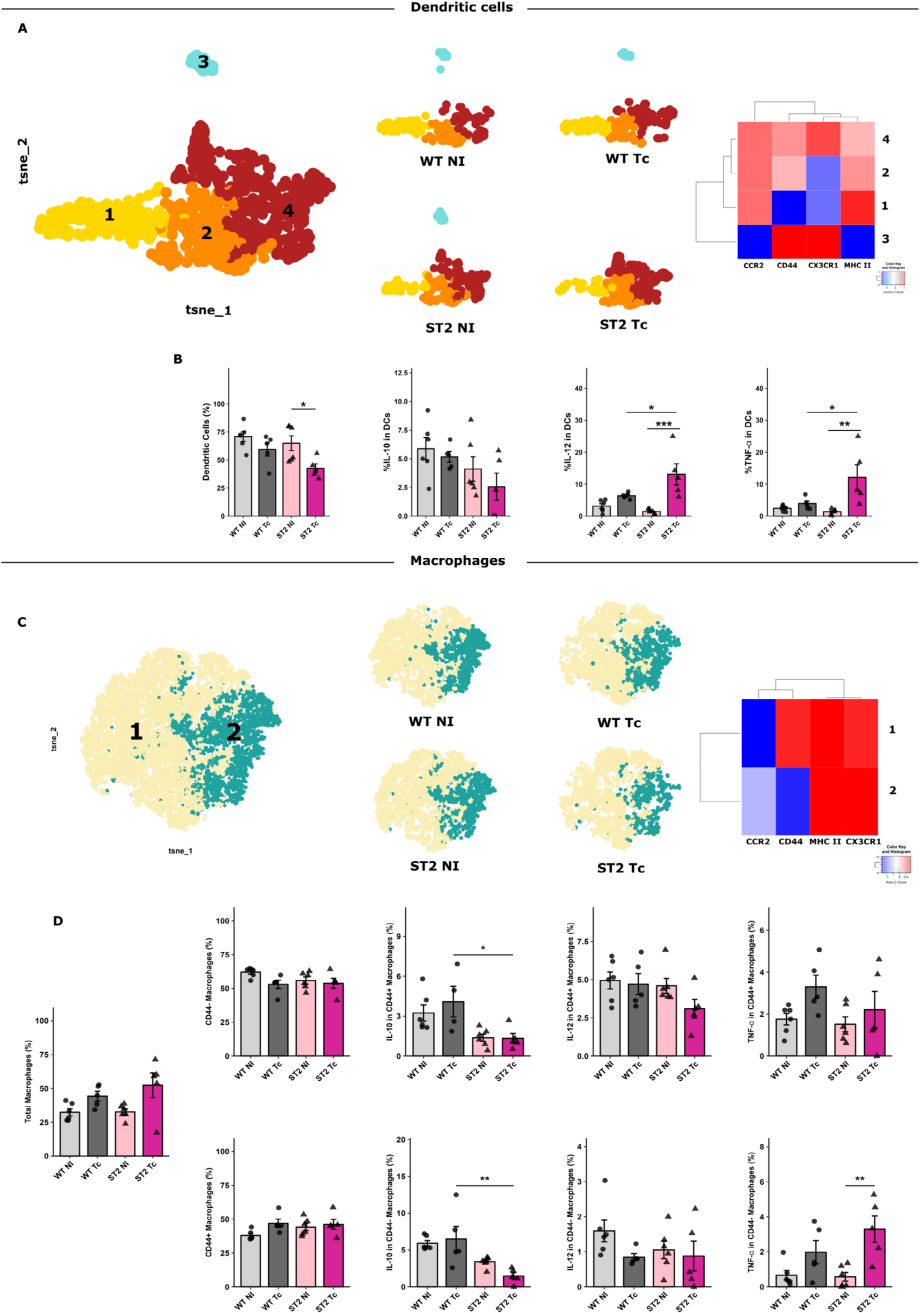
Splenic dendritic cell and macrophage phenotypes during chronic *T. cruzi* infection. (A) t‐SNE analysis of splenic dendritic cells showing four clusters defined by CCR2, CD44, CX3CR1, and MHC II expression. (B) Cytokine expression profiles of dendritic cells, highlighting IL‐10, IL‐12, and TNF. (C) t‐SNE analysis of splenic macrophages identifying CD44+ and CD44‐ subsets. (D) Frequency and cytokine expression profiles of macrophage subsets. Data represent mean ± SEM from *N* = 5–6 mice/group. *p < 0.05, **p < 0.01 (One‐way ANOVA with Tukey's post hoc). ST2^−/−^, ST2‐deficient; WT, wild‐type.

Given the altered monocyte compartment, downstream myeloid lineages were examined. Analysis of DCs identified four clusters defined by differential expression levels of CCR2, CD44, CX3CR1, and MHC II (Figure 8A), arranged along a continuum reflecting recruitment, maturation, tissue homing, and adaptive priming. In WT mice, all four DC states were detected, including low CCR2 and high CX3CR1 clusters consistent with resident conventional DCs (cDCs) that develop independently of monocyte precursors. Notably, this resident DC cluster was not detected in ST2^−/−^ infected mice, which instead showed predominance of CCR2^+^, monocyte‐derived DC subsets (monocyte‐derived DCs; mo‐DCs). The predominance of mo‐DCs in ST2^−/−^ mice may have functional implications, as these cells are typically associated with inflammatory phenotypes and Th1 priming [36, 37, 38].

DCs are a robust source of cytokines during inflammation [39]. Phenotypic analysis demonstrated that DCs from ST2‐deficient mice exhibited increased TNF and IL‐12 production (Figure 8B). In ST2^−/−^ mice, DCs represented a prominent IL‐12‐producing population, consistent with the Th1‐skewed adaptive responses observed in the T‐cell compartment.

Finally, splenic macrophages were examined. Two macrophage populations were identified, primarily distinguished by CD44 expression (Figure 8C). Total macrophage frequencies and subset proportions remained comparable between genotypes (Figure 8D). However, SMs from ST2^−/−^ infected mice displayed reduced IL‐10 expression and increased TNF production, particularly within the CD44^−^ subset. This shift indicates altered macrophage functional polarization despite preserved macrophage abundance.

Collectively, these results indicate that ST2 deficiency is associated with coordinated alterations in myeloid organization and function, including reduced patrolling monocyte representation, increased representation of inflammatory monocyte‐derived DC‐like populations, and altered macrophage cytokine profiles. These changes coincide with enhanced IL‐12 availability and diminished regulatory signatures, occurring alongside the sustained Th1‐associated inflammatory landscape and vascular dysfunction observed in ST2‐deficient mice during *T. cruzi* infection.

## 4 Discussion

Chagas Disease (CD) remains a neglected tropical disease affecting millions, characterized by progressive tissue dysfunction that arises from sustained dysregulation of host inflammatory responses [40, 41]. The clinical trajectory of CD is dictated by the dynamic balance between *T. cruzi* persistence and immune‐mediated tissue damage [42]. High‐dimensional immunophenotyping has demonstrated that chronic *T. cruzi* infection is associated with a persistent state of immune activation, maintained over time. The sustained expression of early activation markers argues against transient immune engagement and instead supports continuous or recurrent immune stimulation [8]. Specifically, a Th1‐associated immune landscape, characterized by elevated IFN‐γ production, frequently correlates with severe disease outcomes, although its precise functional contribution to the multiorgan pathology described here remains to be fully established [43, 44]. Although the IL‐33/ST2 axis has been implicated in coordinating immune regulation across diverse inflammatory contexts, its role in organizing immune–tissue homeostasis during chronic *T. cruzi* infection has remained poorly defined [45, 46, 47].

To directly assess the role of IL‐33/ST2 signaling in integrating immune, metabolic, and behavioral response to *T. cruzi* infection, we conducted a longitudinal analysis of infected ST2‐deficient mice. While ST2^−/−^ mice appeared to maintain body weight and sustained feeding behavior, this apparent clinical stability was misleading. The maintenance of body weight reflected marked splenomegaly and hepatomegaly rather than preserved metabolic homeostasis, whereas sustained feeding behavior likely represented a compensatory response to the increased energetic demands imposed by persistent inflammation and tissue repair processes [48]. Moreover, behavioral indices such as exploratory activity and anhedonia are regulated by neuroimmune circuits that can be modulated by ST2‐dependent pathways, as previously demonstrated in cerebral malaria and other neuroinflammatory contexts [49, 50, 51, 52]. Collectively, these observations indicate that apparent clinical preservation in ST2‐deficient mice masks an underlying failure to restrain immunopathology.

Notably, a robust IL‐17‐mediated response was exclusive to WT mice. Beyond its classical immune functions, IL‐17 serves as a potent modulator of feeding behavior; by signaling through the hypothalamic IL‐17 receptor A (IL‐17RA), it activates hypothalamic neurons to amplify satiety signals. This neuro‐immune circuit, often triggered by systemic inflammation or infection, effectively suppresses food intake and recalibrates metabolic rhythms [53, 54, 55].

At the hepatic level, loss of IL‐33/ST2 signaling was associated with dysregulated inflammatory responses, in which heightened inflammation was dissociated from effective parasite control and accompanied by pronounced metabolic and vascular disturbances. These findings suggest that ST2 signaling contributes to vascular repair mechanisms or limits the hepatic injury previously observed during *T. cruzi* infection [56]. Notably, the biochemical profile of ST2^−/−^ mice revealed a distinct elevation in serum albumin levels. As albumin is a classic negative acute‐phase protein, the synthesis of which is typically downregulated during systemic inflammation, this increase is unlikely to represent an expansion of hepatic synthetic capacity. This interpretation is consistent with relative hyperalbuminemia resulting from hemoconcentration. While speculative, the significant decrease in fecal water content observed in ST2^−/−^ mice may represent an attempt at intestinal water reabsorption, a potential compensatory mechanism in response to persistent vascular leakage [57, 58]. In addition to its role as a biomarker, albumin has emerged as a critical indicator of pathology during *T. cruzi* infection [59]. The failure of the IL‐33/ST2 axis to coordinate this hepato‐biliary–intestinal fluid axis likely exacerbates disease morbidity, mirroring other lethal infection models where hydration status and vascular integrity are the primary determinants of survival [60].

Fibrosis represents the maladaptive outcome of unresolved tissue injury, where reparative processes overshoot and replace functional parenchyma with collagen‐rich scar tissue [61]. In infected ST2^−/−^ mice, persistent hepatic inflammation failed to resolve, resulting in accelerated and disorganized collagen deposition. This phenotype parallels observations in ST2^−/−^ mice infected with *Schistosoma* spp., where the disruption of this pathway led to loose, disorganized extracellular matrix and inappropriate granuloma organization [62, 63]. The cellular mechanism linking IL‐33/ST2 signaling to this fibrotic drive likely involves both direct and indirect pathways. Evidence from *Schistosoma* models demonstrates that IL‐33/ST2 signaling is a fundamental step for the differentiation of quiescent hepatic stellate cells (HSCs) into α‐SMA‐expressing myofibroblasts, and deficient mice fail to upregulate Col III and Col VI mRNA, highlighting the necessity of this axis for professional myofibroblast activation. Moreover, IL‐33 acts as a potent activator of alternatively activated macrophages (M2), which produce profibrotic cytokines like IL‐13 that further amplify HSC activation. The cellular mechanisms potentially linking IL‐33/ST2 signaling to fibrotic remodeling may involve its role in coordinating inflammatory resolution and tissue repair. Recent evidence indicates that the IL‐33/ST2 axis contributes to macrophage–stromal cell communication during chronic inflammation, regulating reparative responses and extracellular matrix organization [64]. In this context, loss of ST2‐mediated regulatory signaling may favor the persistence of a pro‐inflammatory environment and pathological fibrosis [65]. In our model, the observed fibrosis in the absence of ST2 signaling likely reflects this homeostatic failure, where persistent macrophage accumulation and ongoing hepatocellular injury converge to drive chronic hepatic dysfunction. Future studies utilizing lineage‐specific ST2 deletion or ex vivo HSC‐macrophage cocultures will be essential to disentangle the relative contribution of direct stromal signaling versus secondary inflammatory drivers in *T. cruzi* ‐induced liver pathology.

The gut‐liver axis represents a dynamic bidirectional communication network that plays a crucial role in maintaining systemic immune homeostasis and immune surveillance [66]. Within this disrupted circuit, intestinal inflammation in ST2^−/−^ mice was observed alongside hepatic and systemic dysfunction. While ST2 signaling is essential for protective mucus production and epithelial proliferation in helminth infection models [48, 67, 68] its role during protozoan‐induced intestinal pathology remains less well defined. In infected ST2^−/−^ mice, inflammatory responses were intensified, progressing to prolonged intestinal transit times. This pattern mirrors findings from *Toxoplasma* infection models, in which ST2 signaling can potentiate ileitis, supporting a role for IL‐33/ST2 in limiting excessive effector responses in the gut [69]. Morphometric analyses further revealed significant thickening of colonic layers during chronic infection, consistent with reports of smooth muscle hypertrophy, connective tissue expansion, and fibrotic remodeling in chagasic colonic disease [70]. Mucosal thickening likely reflects epithelial hyperplasia and crypt expansion observed in chronic *T. cruzi* infection [71], whereas muscularis hypertrophy with inflammatory infiltrates and enteric nervous system remodeling represents hallmarks of intestinal pathology [72].

The divergence between hepatic and intestinal parasite control in ST2^−/−^ mice highlights the tissue‐specific nature of IL‐33/ST2‐mediated immune coordination. Previous studies have demonstrated that hepatic clearance of *T. cruzi* depends on the activation of Kupffer cells and recruitment of inflammatory monocytes that differentiate into macrophages, thereby contributing to parasite destruction within the liver [16, 73]. This innate response includes the production of reactive oxygen and nitrogen species by phagocytic cells, which plays a critical role in controlling parasite load, and integrates with extrahepatic T cell responses to sustain antimicrobial immunity while preserving hepatocyte metabolic function under inflammatory stress [73, 74]. Our data show that, despite a pronounced Th1‐skewed environment in ST2‐deficient livers, nitric oxide (NO) production was not robustly elevated. This suggests that in the hepatic microenvironment, ST2 signaling is required to effectively couple inflammatory activation with macrophage effector output. Notably, enhanced hepatic parasitism in ST2^−/−^ mice contrasts with observations in *Leishmania donovani* infection, in which IL‐33 deficiency is associated with exaggerated Th1 responses and improved parasite clearance in the liver [75].

Conversely, colonic parasite control relies predominantly on barrier‐associated defenses, including mucus production and epithelial NO, which limit parasite–host interactions at the mucosal surface [18, 76, 77, 78, 79]. In the intestinal compartment, the loss of ST2‐dependent regulatory signals appears to remove a constitutive brake on mucosal inflammatory tone. This removal results in the robust NO production and expanded PAS‐positive mucus area observed in ST2^−/−^ mice, which collectively enhance local parasiticidal activity. Therefore, the opposite outcomes in these two organs are likely driven by distinct local microenvironmental cues: while the liver requires ST2 to orchestrate professional phagocyte effector functions, the colonic mucosa utilizes the absence of ST2‐mediated type 2 regulation to amplify innate barrier defenses.

The behavior of eosinophils further supports this interpretation. Although eosinophil accumulation increased in both the liver and intestine of ST2‐deficient mice, this expansion did not associate with improved parasite clearance. Instead, this observation aligns with emerging evidence indicating that eosinophils in protozoan infections are more closely linked to tissue remodeling and repair than to direct antimicrobial activity. Indeed, eosinophils are increasingly recognized as regulators of tissue remodeling through TGF‐β secretion, matrix metalloproteinase production, and deposition of extracellular matrix components [80]. The precise contribution of eosinophils to tissue remodeling during *T. cruzi* infection therefore warrants further investigation.

At the systemic level, splenic immune dysregulation emerged as a central upstream feature associated with multiorgan pathology in ST2‐deficient mice. This phenotype was characterized by exaggerated expansion of IFN‐γ‐ and TNF‐producing CD4^+^ and CD8^+^ T cells, accompanied by reduced regulatory and IL‐17‐associated responses. Although IFN‐γ is indispensable for parasite control, excessive or unrestrained production is a well‐established driver of *T. cruzi* ‐associated immunopathology [81, 82]. As a major immunological hub, the spleen serves as both a site of immune activation and a reservoir for effector cells that subsequently seed peripheral tissues. Accordingly, this skewed splenic immune landscape likely amplifies organ‐specific inflammation while failing to generate counter‐regulatory responses necessary to limit tissue damage.

Within this dysregulated systemic immune landscape, failure to sustain an IL‐17 associated response in the absence of IL‐33/ST2 signaling emerges as a key integrative node linking immune imbalance, behavioral adaptation, and tissue vulnerability. During *T. cruzi* infection, IL‐17 functions as a regulatory mediator that couples parasite control with restraint of Th1‐driven immunopathology. Consistent with this role, diminished IL‐17 responses have been associated with worse clinical outcomes in both experimental models and human Chagas disease [83, 84, 85]. Mechanistically, IL‐17 signaling limits excessive Th1 polarization and IFN‐γ/TNF production, in part by promoting regulatory neutrophil programs and IL‐10‐mediated feedback loops [84]. In parallel, IL‐17 contributes to mucosal defense by reinforcing epithelial barrier integrity, inducing antimicrobial peptides, and organizing effective neutrophil responses [86]. The reduction in this counter‐regulatory axis in ST2‐deficient mice coincides with sustained Th1‐type responses and progressive tissue injury across multiple organs, suggesting a potential association that warrants further functional dissection.

The marked reduction in patrolling monocytes and the loss of CD44‐associated organization in ST2^−/−^ mice coincide with reactive thrombocytosis and elevated P‐LCC. While patrolling monocytes (Ly6C^low^) are typically associated with endothelial surveillance, their reduction in ST2‐deficient mice parallels the systemic vascular stress observed during chronic infection. In healthy states, patrolling monocytes (Ly6C^low^) act as endothelial scavengers that clear debris and microparticles to preserve vascular integrity [34, 35, 87, 88]. In murine models of atherosclerosis, the absence of this subset exacerbates endothelial damage, underscoring its protective role along the endothelium, including the engulfment of oxidized lipoproteins and the preservation of vessel wall integrity [34, 89, 90, 91].

Furthermore, reduced CD44 expression in ST2‐deficient monocytes may be associated with alterations in myeloid differentiation dynamics, potentially contributing to the inflammatory phenotype observed in the splenic compartment [92]. Under these conditions, monocytes exhibited a phenotypic profile associated with inflammatory activation and increased representation of monocyte‐derived DC‐like populations. characterized by robust IL‐12 and TNF production. This altered myeloid landscape coincided with enhanced Th1/Tc1‐associated responses in the splenic T‐cell compartment. CD44 functions as a signaling scaffold regulating adhesion, migration, survival, and differentiation through interactions with endothelial hyaluronic acid [93, 94]. Evidence from other disease models, including myelofibrosis, unravels the role of CD44^+^ monocytes in mediating extramedullary hematopoiesis by producing hyaluronic acid that recruits hematopoietic stem cells, emphasizing its importance in tissue homeostasis under hematological stress [33]. Besides, in *Toxocara canis* experimental infection, disruption of IL‐33/ST2 signaling was associated with a marked reduction in circulating monocytes, accompanied by extensive hemorrhagic lesions in highly vascularized organs, including the lungs and brain, suggesting a failure of coordinated vascular repair mechanisms [67]. The deficiency of CD44 expression in ST2^−/−^ monocytes is consistent with an impaired patrolling capacity, which may reflect impaired vascular surveillance capacity. This disruption of the vascular‐myeloid interface provides a plausible association for the systemic homeostatic disturbances observed, though direct evidence of endothelial‐monocyte interaction remains to be established through intravital imaging. Furthermore, since the spleen is an important reservoir of cardiac‐homing monocytes [95], the loss of CD44/patrolling monocytes can also result in exacerbated inflammatory cardiomyopathy in this tissue.

We acknowledge that the phenotypic shift observed from patrolling monocytes toward inflammatory mo‐DCs in ST2^−/−^ mice remains to be validated at the transcriptomic level. Nonetheless, our data are consistent with a working model in which IL‐33/ST2 signaling normally provides essential survival or differentiation cues to the Ly6C^low^ patrolling compartment. This hypothesis aligns with observations in CX3CR1‐deficient models, where impaired vascular surveillance leads to the compensatory expansion of inflammatory monocyte‐derived populations [96]. Furthermore, the loss of CD44‐associated organization in ST2‐deficient monocytes may impair downstream signaling pathways, such as ERK1/2 phosphorylation, which has been shown to modulate the differentiation path between macrophages and dendritic cells [97, 98]. Future studies utilizing single‐cell RNA sequencing will be pivotal to determine if these phenotypic alterations constitute a true transcriptional reprogramming event driven directly by the absence of ST2 signaling. Notably, direct functional assessment of monocyte–endothelial interactions through intravital microscopy or flow‐based adhesion assays was not performed in this study and represents a critical methodological gap. Such approaches would be required to confirm that the observed reduction in patrolling monocyte frequency translates to functionally impaired endothelial surveillance.

Finally, it is important to consider that IL‐33/ST2 signaling may interface with MyD88‐dependent pathways, a central adaptor for innate immune signaling previously implicated in chronic inflammation and T‐cell exhaustion [99]. While the present study does not directly investigate these intracellular cascades, the absence of the ST2 regulatory checkpoint could theoretically favor MyD88‐driven pro‐inflammatory amplification, a conceptual framework that warrants targeted investigation in future mechanistic studies.

Emerging evidence highlights the IL‐33/ST2 axis as an essential regulator of tissue homeostasis across diverse parasitic infections, modulating both immunopathogenesis and tissue repair. In murine models of *Schistosoma japonica*, ST2 deficiency accelerates hepatic pathology and disrupts the balance between regulatory T cells and Th17 cells, exacerbating inflammatory damage [100]. Similarly, pharmacological ST2 blockade during alveolar echinococcosis mitigates hepatic fibro‐inflammatory responses, reducing collagen deposition, tissue inhibitor of metalloproteinases‐1 expression, and M2 macrophage accumulation [101]. This aligns with our observation of accelerated fibrotic remodeling and altered myeloid organization in ST2‐deficient livers during *T. cruzi* infection. At the mucosal barrier, the IL‐33 pathway provides protective effects, such as maintaining epithelial integrity during amebic colitis [102] and mediating parasite ejection mechanisms against helminths [103]. The divergent outcomes observed in the liver and colon of our experimental model reflect these highly tissue‐specific roles, where ST2 signaling must simultaneously manage systemic inflammatory networks and local epithelial barrier functions.

The participation of IL‐33 and its receptor ST2 in maintaining this equilibrium may have clinical implications, particularly when evaluating factors associated with increased disease severity [9, 10, 11]. Clinical outcomes in *T. cruzi* infection are heavily influenced by the coexistence of chronic conditions, including diabetes mellitus, obesity, aging, and metabolic syndrome [2]. These metabolic and age‐related disturbances frequently exhibit parallel dysregulation of the IL‐33/ST2 signaling pathway. Specifically, conditions like diabetes, obesity, and metabolic syndrome are associated with disrupted ST2 availability and impaired IL‐33 release [104, 105, 106]. The baseline reduction of ST2 function in these populations may compromise the host's capacity to engage the regulatory pathways identified in our study, leading to unrestrained Th1 polarization and accelerated tissue damage upon *T. cruzi* infection. Consequently, diminished ST2 signaling may serve as a contributing mechanism linking these chronic comorbidities to the development of severe Chagasic cardiomyopathy and hepato‐intestinal complications. Tracking the longitudinal dynamics of IL‐33 and ST2 could therefore provide measurable parameters for evaluating host immune competence and tissue vulnerability during interventional assays, prolonged treatments, and vaccine development against Chagas disease.

While the current study identifies a consistent association between ST2 deficiency, elevated IFN‐γ production, and multiorgan pathology, we acknowledge that the lack of direct functional modulation, such as in vivo IFN‐γ blockade, precludes a definitive causal link. Future investigations utilizing targeted pathway inhibition will be instrumental in determining whether the observed Th1‐type expansion is a primary driver of tissue damage or a concomitant feature of a broader homeostatic collapse.

We also acknowledge several limitations in the present study. First, while high‐dimensional spectral immunophenotyping was performed in the spleen to provide an overview of the systemic immune landscape, this does not necessarily capture the tissue‐resident immune dynamics occurring directly within the liver and intestine, the primary sites of pathology. Future studies incorporating organ‐specific immune profiling are required to determine if the myeloid and lymphoid alterations observed systemically are mirrored or amplified locally. Furthermore, the descriptive nature of our findings, supported by indirect measures such as enzymatic activities and Evans blue permeability, precludes definitive causal conclusions. The absence of direct interventional experiments, such as in vivo IFN blockade or more specific epithelial barrier assays, such as FITC‐dextran extravasation or molecular characterization of cell–cell junctions (e.g., occludin, claudins), means that the proposed mechanistic framework should be interpreted as a series of hypothesis‐generating associations rather than established causal links.

Collectively, our findings position IL‐33/ST2 signaling as a critical integrator of immune regulation, vascular homeostasis, and metabolic adaptation during chronic parasitic infection. ST2 signaling integrates tissue‐specific immune restraint with the preservation of endothelial integrity and systemic homeostasis. The absence of ST2 signaling was associated with a maladaptive state in which early clinical preservation masks progressive immune, vascular, and metabolic dysfunction. This phenotype was accompanied by disruption of vascular‐associated myeloid organization, which is characterized by a loss of CD44‐dependent patrolling monocytes, skewed myeloid differentiation, and unchecked Th1 amplification. These conditions ultimately predispose the body to organ‐specific pathology and chronic tissue injury in *T. cruzi* infection.

## 5 Conclusion

Our study demonstrates that disruption of IL‐33/ST2 signaling is associated with profound alterations in systemic immunity and hepato‐intestinal homeostasis during experimental *T. cruzi* infection. ST2 deficiency coincided with amplified inflammatory cytokine responses, altered myeloid and lymphoid immune organization, increased hepatic pathology, and progressive intestinal remodeling. Although the mechanisms underlying these observations remain to be fully elucidated, our findings support a role for the IL‐33/ST2 axis in the regulation of immune and tissue responses during Chagas disease. These results provide a foundation for future mechanistic studies exploring IL‐33/ST2‐targeted interventions in chronic parasitic infections.

## Author Contributions

Marcelo Eduardo Cardozo, Fabiana Simão Machado, Ricardo Toshio Fujiwara developed the overall concept and designed the experiments. Marcelo Eduardo Cardozo, Tatyane Martins Cirilo, José Bryan da Rocha Rihs, Isabela de Brito Duval, Ana Rafaela Antunes‐Porto, Luisa Vitor do Braga do Amaral, Fernando Bento Rodrigues Oliveira, Mayra Fernanda Ricci, Laura Lis de Oliveira Santos, Lívia Fernanda Dias Santana, Luiza Pinheiro Silva, Chiara Cássia Oliveira Amorim, Gabriela Gomes Monteiro Lemos, Getulío Mota e Silva Junior, Izabela da Silva Oliveira, Jorge Lucas Nascimento Souza, Ana Laura Grossi de Oliveira, Marina Possa dos Reys, Geovanni Dantas Cassali performed experiments and analyzed the data. Geovanni Dantas Cassali, Luisa Mourão Dias Magalhães, Lilian Lacerda Bueno, Fabiana Simão Machado, Ricardo Toshio Fujiwara provided essential tools and expertise. All authors read and approved the final manuscript.

## Funding

This study received partial financial support from the Fundação de Amparo à Pesquisa do Estado de Minas Gerais (FAPEMIG), Brazil (Grants APQ‐02628‐24, APQ‐04574‐25, APQ‐01816‐23, CBB APQ‐00766‐18, RED‐00096‐22, and RED‐00067‐23). Additional support was provided by the National Institute for Science and Technology (INCT) in Dengue and Host–Microbial Interactions (CNPq Grant 408527/2024‐2) and by INCT Mucosas e Pele (CNPq Grant 408484/2024‐1). This research was also funded by the Brazilian National Research Council (CNPq) (Grants 421392/2018‐5 and 302491/2017‐1) and by the Pró‐Reitoria de Pesquisa of the Universidade Federal de Minas Gerais (UFMG) to support research inputs. M.E.C. acknowledges the PhD fellowship provided by the Coordenação de Aperfeiçoamento de Pessoal de Nível Superior (CAPES) through the Postgraduate Program in Infectious Diseases and Tropical Medicine at UFMG. LLB (CNPq Grant 310311/2023‐3), FSM (CNPq Grant 307828/2022‐0), and RTF (CNPq Grant 305514/2022‐9) are research fellows of CNPq. GDC is supported by FAPEMIG through the Rede Mineira de Pesquisa Translacional em Imunobiológicos e Biofármacos no Câncer (REMITRIBIC; Grant RED‐00031‐21), and ALGO received a postdoctoral fellowship from FAPEMIG (APD‐01254/25). The funding agencies had no role in the study design; data collection, analysis, or interpretation; decision to publish; payment of publication fees; or preparation of the manuscript.

## Ethics Statement

All animal experiments were performed according to the Animal Ethics Committee of UFMG (12/2024). All experimental procedures, the number of animals, and their risk of suffering from treatments were minimized.

## Conflicts of Interest

The authors declare no conflicts of interest.

## Supporting information


**Table S1:** Comprehensive Overview of Erythrocyte and Platelet Indices Evaluated during Experimental *T. cruzi* Infection.
**Figure S1:** ST2‐deficient mice exhibit altered organ‐to‐body weight ratios across acute and chronic phases of *T. cruzi* infection. (A) Kinetic analysis of organ weights (Liver, Spleen, Heart, Kidney, and Lung) expressed as a percentage of total body weight in Wild‐type (WT) and ST2‐deficient (ST2) mice. Measurements were taken at 0, 7, 20, and 100 days postinfection (DPI) with *T. cruzi* (Tc) or in noninfected (NI) controls. Data are expressed as mean ± SEM (*n* = 5–6). ****p < 0.0001. Two‐way ANOVA with Tukey's post hoc. ST2^−/−^, ST2‐deficient; WT, wild‐type.
**Figure S2:** Histological characterization of hepatic inflammatory infiltrates and fibrotic remodeling. Representative photomicrographs of liver sections from Wild‐type (WT) and ST2‐deficient (ST2^−/−^) mice at 0, 7, 20, and 100 days postinfection (DPI) with *T. cruzi* . (A) Hematoxylin & Eosin (H&E) staining at increased magnification facilitates the identification of cellular morphology within inflammatory foci. White circles highlight multifocal aggregates. (B) Masson's Trichrome staining provides detailed visualization of collagen deposition (blue staining), particularly highlighting the accelerated and disorganized fibrotic remodeling in ST2^−/−^ mice during the transition to chronicity. Scale bar: 100 μm. ST2^−/−^, ST2‐deficient; WT, wild‐type.
**Figure S3:** Kinetics of systemic cytokine profiles during experimental *Trypanosoma cruzi* infection in WT and ST2^−/−^ mice. Splenic protein concentrations of Th1, Th2, Th17, and innate inflammatory cytokines (IFN‐γ, TNF, IL‐12, IL‐6, IL‐2, IL‐17, IL‐10, and IL‐4) were quantified at predefined time points (0, 7, 20, and 100 days postinfection). Measurements for IFN‐γ, TNF, IL‐6, IL‐2, IL‐17, IL‐10, and IL‐4 were performed using a cytometric bead array (CBA) Th1/Th2/Th17 kit, while IL‐12 levels were determined via sandwich enzyme‐linked immunosorbent assay (ELISA). Data are presented as a heatmap of mean *Z*‐scores to illustrate temporal variance and relative shifts across genotypes. Bars or cells represent mean values from *N* = 5–6 biological replicates per group. *p < 0.05, **p < 0.01, ***p < 0.001 versus corresponding wild‐type (WT) controls at each respective time point (Two‐way ANOVA followed by Tukey's post hoc test). ST2^−/−^, ST2‐deficient.
**Figure S4:** Representative flow cytometry gating strategy for T lymphocyte effector function. Hierarchical gating strategy for the identification of cytokine‐producing CD4+ and CD8+ T cells. Sequential gating flow used to identify intracellular cytokine expression in splenic T cells. Briefly, total cells were first gated by size and granularity (FSC‐A vs. SSC‐A), followed by singlet discrimination (SSC‐H vs. SSC‐A). Viable cells were identified by exclusion of a fixable viability dye. Within the live population, CD4+ (P1) and CD8+ (P2) T cell subsets were defined. Representative density plots show the quadrant gating for IFN‐γ, IL‐17 and TNF expression within the respective T cell compartments. These gates were used to calculate the frequencies and ratios presented in Figures S3–S6.
**Figure S5:** Direct visualization of cytokine distribution profiles in splenic T cells during *T. cruzi* infection. Plots displaying the distribution of Mean Fluorescence Intensity (MFI) for IFNg, IL‐10, IL‐17, IL‐4, and TNF within (A) CD4+ and (B) CD8+ T cell compartments. Each density curve represents the distribution of cytokine expression levels across individual samples within the experimental groups: Wild‐type (WT) and ST2‐deficient (ST2^−/−^) mice. The overlapping peaks illustrate the population shifts and functional heterogeneity associated with ST2 deficiency. Data are derived from high‐dimensional spectral flow cytometry. MFI, mean fluorescence intensity; ST2^−/−^: ST2‐deficient; WT, wild‐type.
**Figure S6:** Skewed Th1/Th2/Th17 balance in acute CD4+ T cells of ST2‐deficient mice. Ratios of pro‐inflammatory versus anti‐inflammatory/regulatory cytokines in splenic CD4+ T lymphocytes at the acute stage of infection. (A–C) Ratios of TNF relative to IL‐4, IL‐10, and IL‐17. (D–F) Ratios of IFN‐γ relative to IL‐4, IL‐10, and IL‐17. Data represent individual mice and mean ± SEM. Data represent mean ± SEM from *N* = 5–6 mice/group. *p < 0.05, **p < 0.01 (One‐way ANOVA with Tukey's post hoc). ST2^−/−^, ST2‐deficient; WT, wild‐type.
**Figure S7:** Enhanced inflammatory effector ratios in CD8+ T cells of ST2‐deficient mice during acute infection. Analysis of cytokine production balance in CD8+ T lymphocytes. (A) IFN‐γ/IL‐10, (B) TNF/IL‐17, (C) TNF/IL‐10, and (D) IFN‐γ/IL‐17 ratios. Data represent mean ± SEM from *N* = 5–6 mice/group. *p < 0.05, **p < 0.01 (One‐way ANOVA with Tukey's post hoc). ST2^−/−^, ST2‐deficient; WT, wild‐type.
**Figure S8:** ST2‐deficiency leads to sustained and exacerbated pro‐inflammatory CD4+ T cell ratios in chronic Chagas disease. Cytokine ratios in splenic CD4+ T cells at 100 DPI. (A–C) TNF ratios and (D–F) IFN‐γ ratios relative to IL‐4, IL‐10, and IL‐17 in the chronic phase. Data represent mean ± SEM from *N* = 5–6 mice/group. *p < 0.05, **p < 0.01 (One‐way ANOVA with Tukey's post hoc). ST2^−/−^, ST2‐deficient; WT, wild‐type.
**Figure S9:** Persistent Th1‐like bias in CD8+ T cells of ST2‐deficient mice during the chronic stage of *T. cruzi* infection. Evaluation of CD8+ T cell cytokine ratios at 100 DPI. (A–F) Comprehensive analysis of inflammatory balance. ST2 KO mice maintain significantly higher TNF and IFN‐γ ratios relative to IL‐4 and IL‐17 compared to WT mice. Data represent mean ± SEM from *N* = 5–6 mice/group. *p < 0.05, **p < 0.01 (One‐way ANOVA with Tukey's post hoc). ST2^−/−^, ST2‐deficient; WT, wild‐type.
**Figure 10:** Multiparametric flow cytometry identification of monocyte and macrophage subpopulations. Representative gating strategy for the characterization of myeloid compartments in the spleen. Total leukocytes were gated for size/granularity and singlets. CD45+ viable cells were selected for lineage analysis. CD11b + cells were further subdivided based on CX3CR1, Ly6C, and MHC II expression to identify: P1 (dendritic cells), P2 monocytes, P3/P4 monocyte subsets (classical Ly6C^high^ and patrolling Ly6C^low^), and P5‐P7 (total macrophages and subsets based on CD44 activation markers).

## Data Availability

The data that support the findings of this study are available from the corresponding author upon reasonable request.
