# Supplementary material for: IL‐33/ST2 Signaling Sustains Hepato‐Intestinal Homeostasis by Orchestrating Vascular Surveillance and Immune Regulatory Circuits During Experimental Trypanosoma cruzi Infection
Source: FASEB J. 2026 Jul 5;40(13):e72116. doi: 10.1096/fj.202600565RR (PMC13333159; doi:10.1096/fj.202600565RR)
Supplement: Supplementary file 1 — Table S1: Comprehensive Overview of Erythrocyte and Platelet Indices Evaluated during Experimental T. cruzi Infection. Figure S1: ST2‐deficient mice exhibit altered organ‐to‐body weight ratios across acute and chronic phases of T. cruzi infection. (A) Kinetic analysis of organ weights (Liver, Spleen, Heart, Kidney, and Lung) expressed as a percentage of total body weight in Wild‐type (WT) and ST2‐deficient (ST2) mice. Measurements were taken at 0, 7, 20, and 100 days postinfection (DPI) with T. cruzi (Tc) or in noninfected (NI) controls. Data are expressed as mean ± SEM (n = 5–6). ****p < 0.0001. Two‐way ANOVA with Tukey's post hoc. ST2−/−, ST2‐deficient; WT, wild‐type. Figure S2: Histological characterization of hepatic inflammatory infiltrates and fibrotic remodeling. Representative photomicrographs of liver sections from Wild‐type (WT) and ST2‐deficient (ST2−/−) mice at 0, 7, 20, and 100 days postinfection (DPI) with T. cruzi . (A) Hematoxylin & Eosin (H&E) staining at increased magnification facilitates the identification of cellular morphology within inflammatory foci. White circles highlight multifocal aggregates. (B) Masson's Trichrome staining provides detailed visualization of collagen deposition (blue staining), particularly highlighting the accelerated and disorganized fibrotic remodeling in ST2−/− mice during the transition to chronicity. Scale bar: 100 μm. ST2−/−, ST2‐deficient; WT, wild‐type. Figure S3: Kinetics of systemic cytokine profiles during experimental Trypanosoma cruzi infection in WT and ST2−/− mice. Splenic protein concentrations of Th1, Th2, Th17, and innate inflammatory cytokines (IFN‐γ, TNF, IL‐12, IL‐6, IL‐2, IL‐17, IL‐10, and IL‐4) were quantified at predefined time points (0, 7, 20, and 100 days postinfection). Measurements for IFN‐γ, TNF, IL‐6, IL‐2, IL‐17, IL‐10, and IL‐4 were performed using a cytometric bead array (CBA) Th1/Th2/Th17 kit, while IL‐12 levels were determined via sandwich enzyme‐linked immunosorbent assay (ELISA). Dat [file FSB2-40-e72116-s001.docx]

**IL-33/ST2 Signaling Sustains Hepato-Intestinal Homeostasis by Orchestrating Vascular Surveillance and Immune Regulatory Circuits during Experimental *Trypanosoma cruzi* Infection**

Marcelo Eduardo Cardozo^1,2^, Tatyane Martins Cirilo^1^, José Bryan da Rocha Rihs^1^, Jorge Lucas Nascimento Souza^1^, Isabela de Brito Duval^1^, Ana Rafaela Antunes-Porto^1^, Luisa Vitor Braga do Amaral^1^, Fernando Bento Rodrigues Oliveira^3^, Mayra Fernanda Ricci^3^, Laura Lis de Oliveira Santos^3^, Lívia Fernanda Dias Santana^3^, Luiza Pinheiro Silva^3^, Chiara Cássia Oliveira Amorim^1^, Gabriela Gomes Monteiro Lemos^1^, Getulío Mota e Silva Junior^1^, Izabela da Silva Oliveira^1^, Marina Possa dos Reys^4^, Ana Laura Grossi de Oliveira^1,2^, Geovanni Dantas Cassali^4^, Luisa Mourão Dias Magalhães^5,6^, Lilian Lacerda Bueno^1,2,6^, Fabiana Simão Machado^2,3*^, Ricardo Toshio Fujiwara^1,2,6*^.

^1^ Laboratory of Immunobiology and Parasite Control, Institute of Biological Sciences, Universidade Federal de Minas Gerais, Belo Horizonte, Brazil,

^2^ Post-graduation Program in Health Sciences: Infectious Diseases and Tropical Medicine, Faculdade de Medicina, Universidade Federal de Minas Gerais, Belo Horizonte, Brazil,

^3^ Laboratory of Immunoregulation of Infectious Diseases, Institute of Biological Sciences, Universidade Federal de Minas Gerais, Belo Horizonte, Brazil,

^4^ Laboratory of Comparative Pathology, Institute of Biological Sciences, Institute of Biological Sciences, Universidade Federal de Minas Gerais, Belo Horizonte, Brazil,

^5^ Laboratory of Interactions in ImmunoParasitology, Institute of Biological Sciences, Institute of Biological Sciences, Universidade Federal de Minas Gerais, Belo Horizonte, Brazil

^6^ Institute of Research in Mucosa and Skin (INCT Mucosa and Skin), Universidade Federal de Minas Gerais, Belo Horizonte, Brazil

**Correspondence**

*Prof. Ricardo Toshio Fujiwara

Laboratory of Immunobiology and Parasite Control, Institute of Biological Sciences, Federal University of Minas Gerais, 6627, Antônio Carlos Avenue, Pampulha Campus: Building E4/Room 173, Belo Horizonte, Brazil.

E-mail address: rtfujiwara@gmail.com (R.T. Fujiwara).

ORCID: https://orcid.org/0000-0002-4713-575X

*Prof. Fabiana Simão Machado

Laboratory of Immunoregulation of Infectious Diseases, Institute of Biological Sciences, Universidade Federal de Minas Gerais, Belo Horizonte, Brazil, 6627, Antônio Carlos Avenue, Pampulha Campus: Building O4/Room 190, Belo Horizonte, Brazil.

E-mail address: machadofs@gmail.com (F.S. Machado).

ORCID: https://orcid.org/0000-0001-9272-5209

**Supplementary Information**

**Supplementary Table 1. Comprehensive Overview of Erythrocyte and Platelet Indices Evaluated during Experimental *T. cruzi* Infection.**

| Abbreviation | Description | Clinical meaning | Unit |
| --- | --- | --- | --- |
| MCV | [Mean](https://dictionary.cambridge.org/pt/dicionario/ingles-portugues/mean) corpuscular [volume](https://dictionary.cambridge.org/pt/dicionario/ingles-portugues/volume) | Indicates the average red blood cell size. | fL |
| MCH | [Mean](https://dictionary.cambridge.org/pt/dicionario/ingles-portugues/mean) corpuscular [hemoglobin](https://dictionary.cambridge.org/pt/dicionario/ingles-portugues/hemoglobin) | Reflects the mean hemoglobin content per erythrocyte. | pg |
| RDW-SD | S[tandard](https://dictionary.cambridge.org/pt/dicionario/ingles-portugues/standard) d[eviation](https://dictionary.cambridge.org/pt/dicionario/ingles-portugues/deviation) of RDW (d[istribution](https://dictionary.cambridge.org/pt/dicionario/ingles-portugues/distribution) of erythrocytes) | Quantifies anisocytosis (erythrocyte size variation). | fL |
| PDW | [Platelet](https://dictionary.cambridge.org/pt/dicionario/ingles-portugues/platelet) d[istribution](https://dictionary.cambridge.org/pt/dicionario/ingles-portugues/distribution) a[mplitude](https://dictionary.cambridge.org/pt/dicionario/ingles-portugues/amplitude) | Indicates variation in platelet size, useful for evaluating platelet disorders. | fL |
| MPV | [Mean](https://dictionary.cambridge.org/pt/dicionario/ingles-portugues/mean) [platelet](https://dictionary.cambridge.org/pt/dicionario/ingles-portugues/platelet) [volume](https://dictionary.cambridge.org/pt/dicionario/ingles-portugues/volume) | Indicates the average platelet size. | fL |
| MCHC | Mean corpuscular concentration hemoglobin | Indicates the average hemoglobin concentration in red blood cells. | g/dL |
| RBC | R[ed](https://dictionary.cambridge.org/pt/dicionario/ingles-portugues/red) [blood](https://dictionary.cambridge.org/pt/dicionario/ingles-portugues/blood) [cell](https://dictionary.cambridge.org/pt/dicionario/ingles-portugues/cell) (erythrocytes) [count](https://dictionary.cambridge.org/pt/dicionario/ingles-portugues/count) | Number of circulating red blood cells. | x10^6^/μL |
| HGB | H[emoglobin](https://dictionary.cambridge.org/pt/dicionario/ingles-portugues/hemoglobin) | Indicates the blood’s oxygen-carrying capacity. | g/dL |
| HCT | Hematocrit | Shows the proportion of red blood cells in blood. | % |
| RDW-CV | [Coefficient](https://dictionary.cambridge.org/pt/dicionario/ingles-portugues/coefficient) [of](https://dictionary.cambridge.org/pt/dicionario/ingles-portugues/of) [variation](https://dictionary.cambridge.org/pt/dicionario/ingles-portugues/variation) [of](https://dictionary.cambridge.org/pt/dicionario/ingles-portugues/of) RDW | Shows the variation in red blood cell size. | % |
| PLT | [Platelet](https://dictionary.cambridge.org/pt/dicionario/ingles-portugues/platelet) [count](https://dictionary.cambridge.org/pt/dicionario/ingles-portugues/count) | Shows the number of platelets, used to evaluate bleeding risk and platelet disorders. | x10^3^/μL |
| PCT | Plateletcrit | Represents the percentage of blood volume occupied by platelets (analogous to hematocrit). | % |
| P_LCC | [Giant](https://dictionary.cambridge.org/pt/dicionario/ingles-portugues/giant) platelets | Quantitative measure of large platelet sub-populations, indicating altered megakaryopoiesis. | x10^3^/μL |


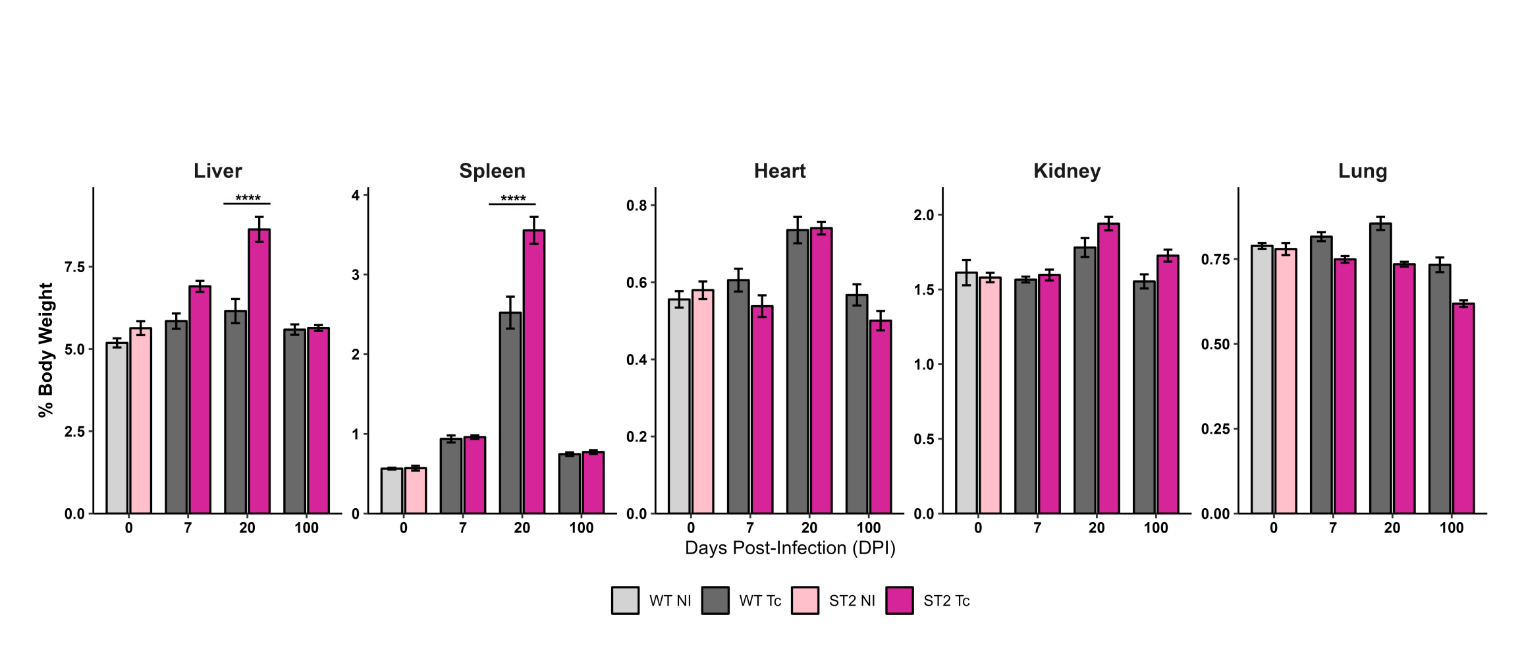


**Supplementary figure 1.** ST2-deficient mice exhibit altered organ-to-body weight ratios across acute and chronic phases of *T. cruzi* infection. (A) Kinetic analysis of organ weights (Liver, Spleen, Heart, Kidney, and Lung) expressed as a percentage of total body weight in Wild-type (WT) and ST2-deficient (ST2) mice. Measurements were taken at 0, 7, 20, and 100 days post-infection (DPI) with *T. cruzi* (Tc) or in non-infected (NI) controls. Data are expressed as mean ± SEM (n = 5-6). ****p < 0.0001. Two-way ANOVA with Tukey’s post-hoc. WT: wild‑type; ST2^-/-^ : ST2‑deficient.


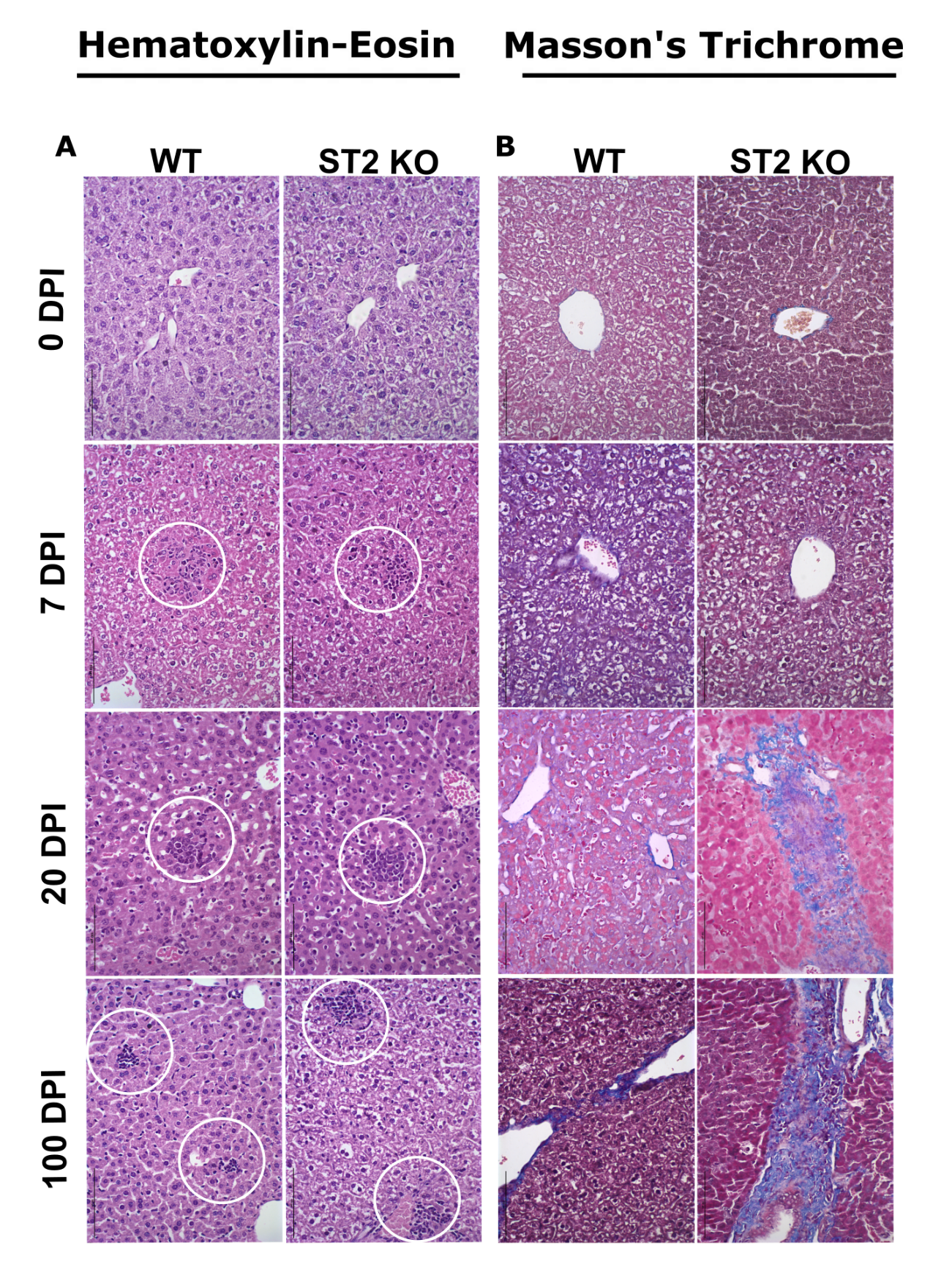


**Supplementary figure 2. Histological characterization of hepatic inflammatory infiltrates and fibrotic remodeling**. Representative photomicrographs of liver sections from Wild-type (WT) and ST2-deficient (ST2^-/-^) mice at 0, 7, 20, and 100 days post-infection (DPI) with *T. cruzi*. (A) Hematoxylin & Eosin (H&E) staining at increased magnification facilitates the identification of cellular morphology within inflammatory foci. White circles highlight multifocal aggregates. (B) Masson’s Trichrome staining provides detailed visualization of collagen deposition (blue staining), particularly highlighting the accelerated and disorganized fibrotic remodeling in ST2^-/-^ mice during the transition to chronicity. Scale bar: 100 µm. WT: wild-type; ST2^-/-^: ST2-deficient.


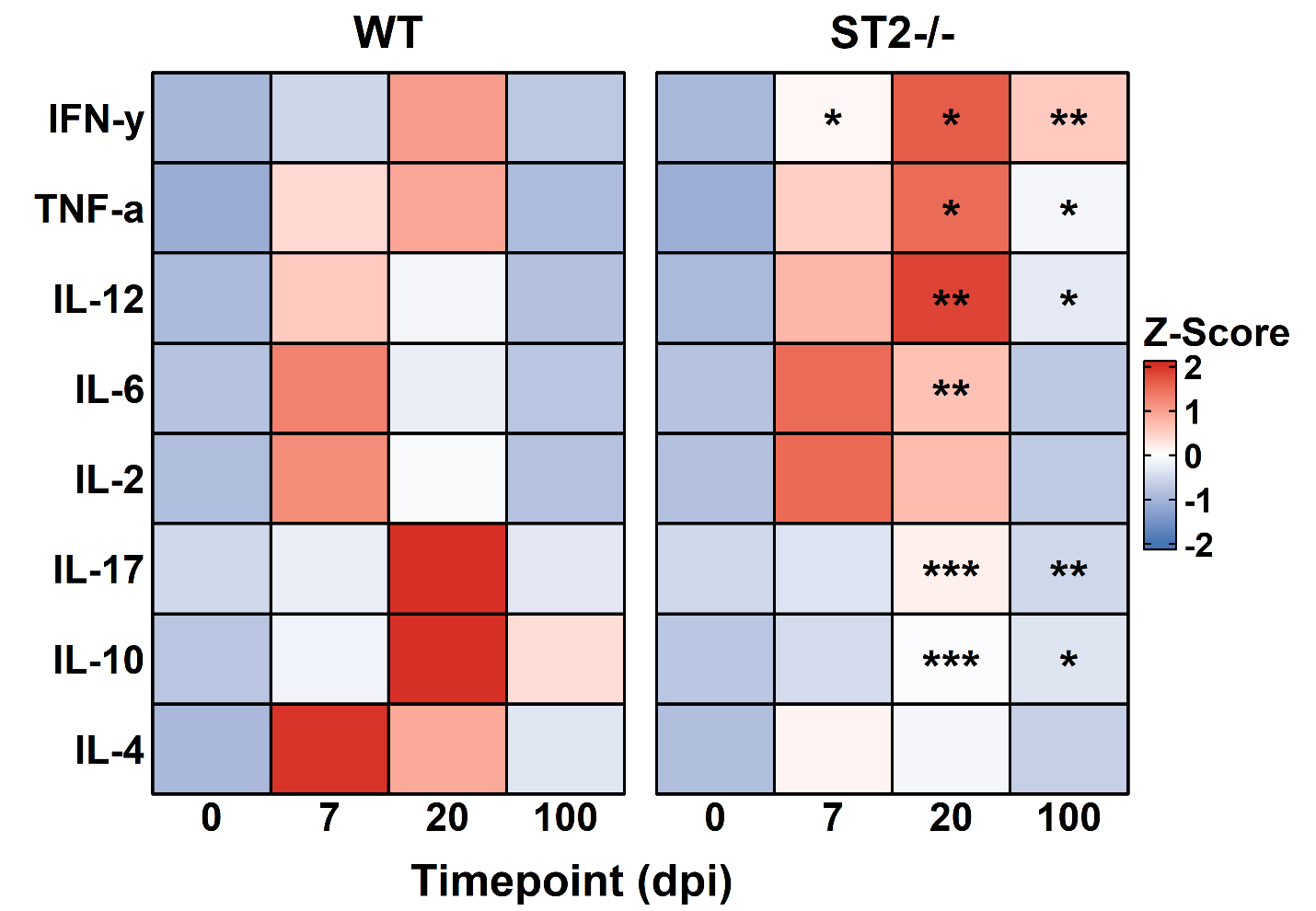


**Supplementary Figure S3. Kinetics of systemic cytokine profiles during experimental *Trypanosoma cruzi* infection in WT and ST2^⁻/⁻^ mice.** Splenic protein concentrations of Th1, Th2, Th17, and innate inflammatory cytokines (IFN-γ, TNF, IL-12, IL-6, IL-2, IL-17, IL-10, and IL-4) were quantified at predefined time points (0, 7, 20, and 100 days post-infection). Measurements for IFN-γ, TNF, IL-6, IL-2, IL-17, IL-10, and IL-4 were performed using a cytometric bead array (CBA) Th1/Th2/Th17 kit, while IL-12 levels were determined via sandwich enzyme-linked immunosorbent assay (ELISA). Data are presented as a heatmap of mean Z-scores to illustrate temporal variance and relative shifts across genotypes. Bars or cells represent mean values from N = 5-6 biological replicates per group. *p < 0.05, * *p < 0.01, * * *p < 0.001 versus corresponding wild-type (WT) controls at each respective time point (Two-way ANOVA followed by Tukey's post-hoc test). ST2^⁻/⁻^: ST2-deficient.


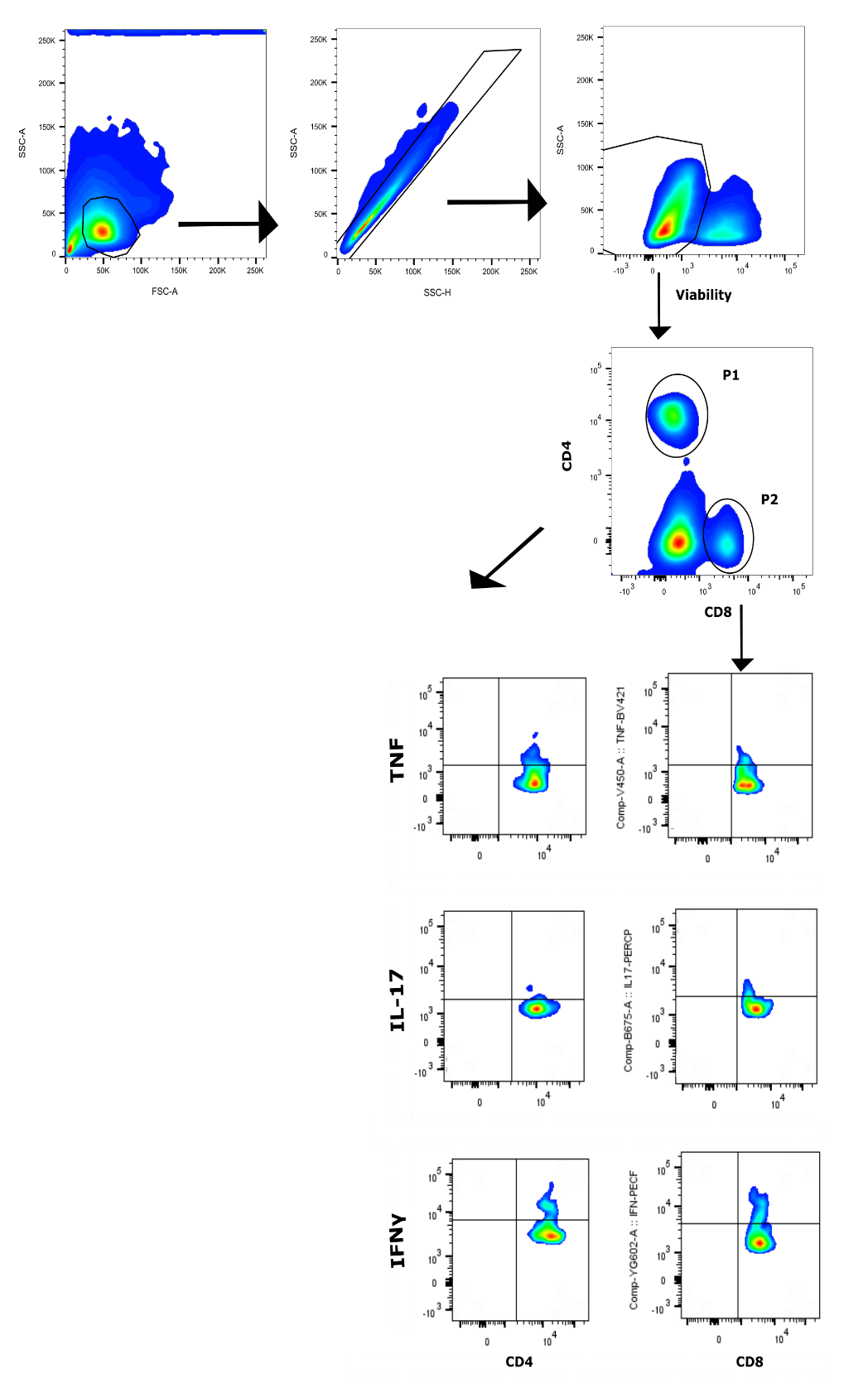


**Supplementary figure 4. Representative flow cytometry gating strategy for T lymphocyte effector function.** Hierarchical gating strategy for the identification of cytokine-producing CD4+ and CD8+ T cells. Sequential gating flow used to identify intracellular cytokine expression in splenic T cells. Briefly, total cells were first gated by size and granularity (FSC-A vs. SSC-A), followed by singlet discrimination (SSC-H vs. SSC-A). Viable cells were identified by exclusion of a fixable viability dye. Within the live population, CD4+ (P1) and CD8+ (P2) T cell subsets were defined. Representative density plots show the quadrant gating for IFN-γ, IL-17 and TNF expression within the respective T cell compartments. These gates were used to calculate the frequencies and ratios presented in Figures S3–S6.


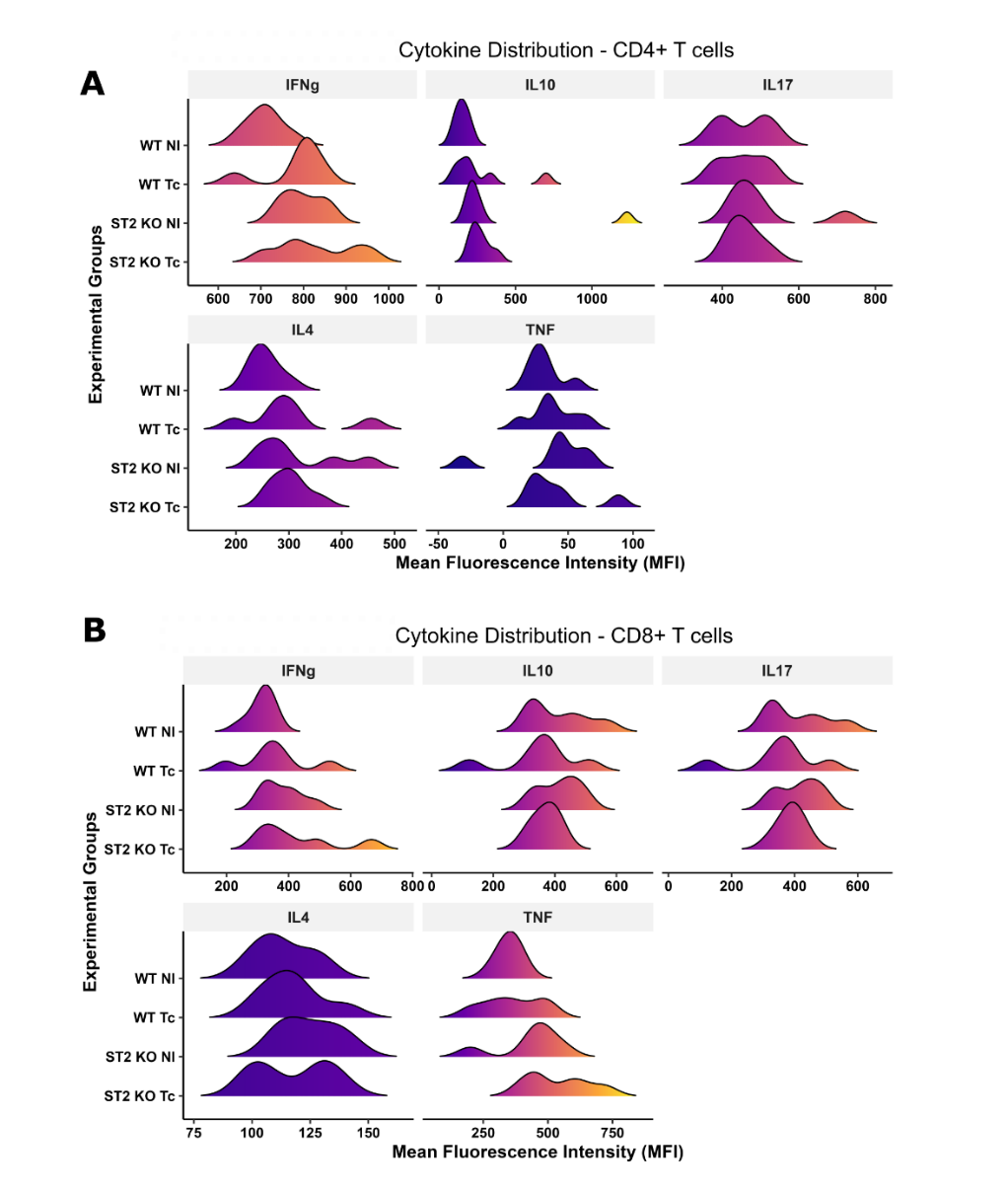


**Supplementary figure 5. Direct visualization of cytokine distribution profiles in splenic T cells during *T. cruzi* infection.** Plots displaying the distribution of Mean Fluorescence Intensity (MFI) for IFNg, IL-10, IL-17, IL-4, and TNF within (A) CD4+ and (B) CD8+ T cell compartments. Each density curve represents the distribution of cytokine expression levels across individual samples within the experimental groups: Wild-type (WT) and ST2-deficient (ST2^-/-^) mice. The overlapping peaks illustrate the population shifts and functional heterogeneity associated with ST2 deficiency. Data are derived from high-dimensional spectral flow cytometry. WT: wild-type; ST2^-/-^: ST2-deficient; MFI: mean fluorescence intensity.

**
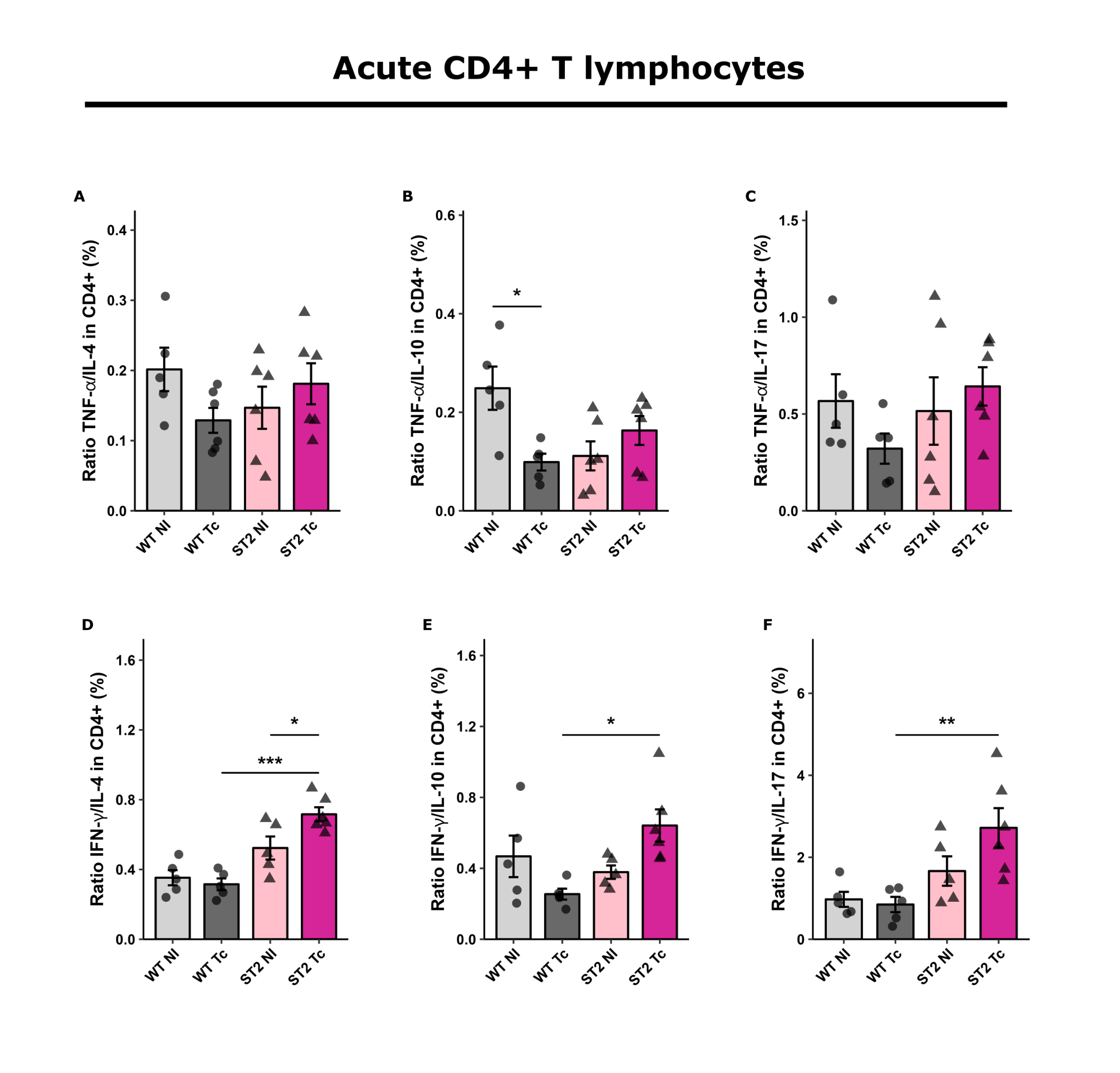
**

**Supplementary figure 6. Skewed Th1/Th2/Th17 balance in acute CD4+ T cells of ST2-deficient mice.** Ratios of pro-inflammatory vs. anti-inflammatory/regulatory cytokines in splenic CD4+ T lymphocytes at the acute stage of infection. (A-C) Ratios of TNF relative to IL-4, IL-10, and IL-17. (D-F) Ratios of IFN-γ relative to IL-4, IL-10, and IL-17. Data represent individual mice and mean ± SEM. Data represent mean ± SEM from N = 5–6 mice/group. *p < 0.05, **p < 0.01 (One‑way ANOVA with Tukey’s post‑hoc). WT: wild‑type; ST2^-/-^ : ST2‑deficient.

**
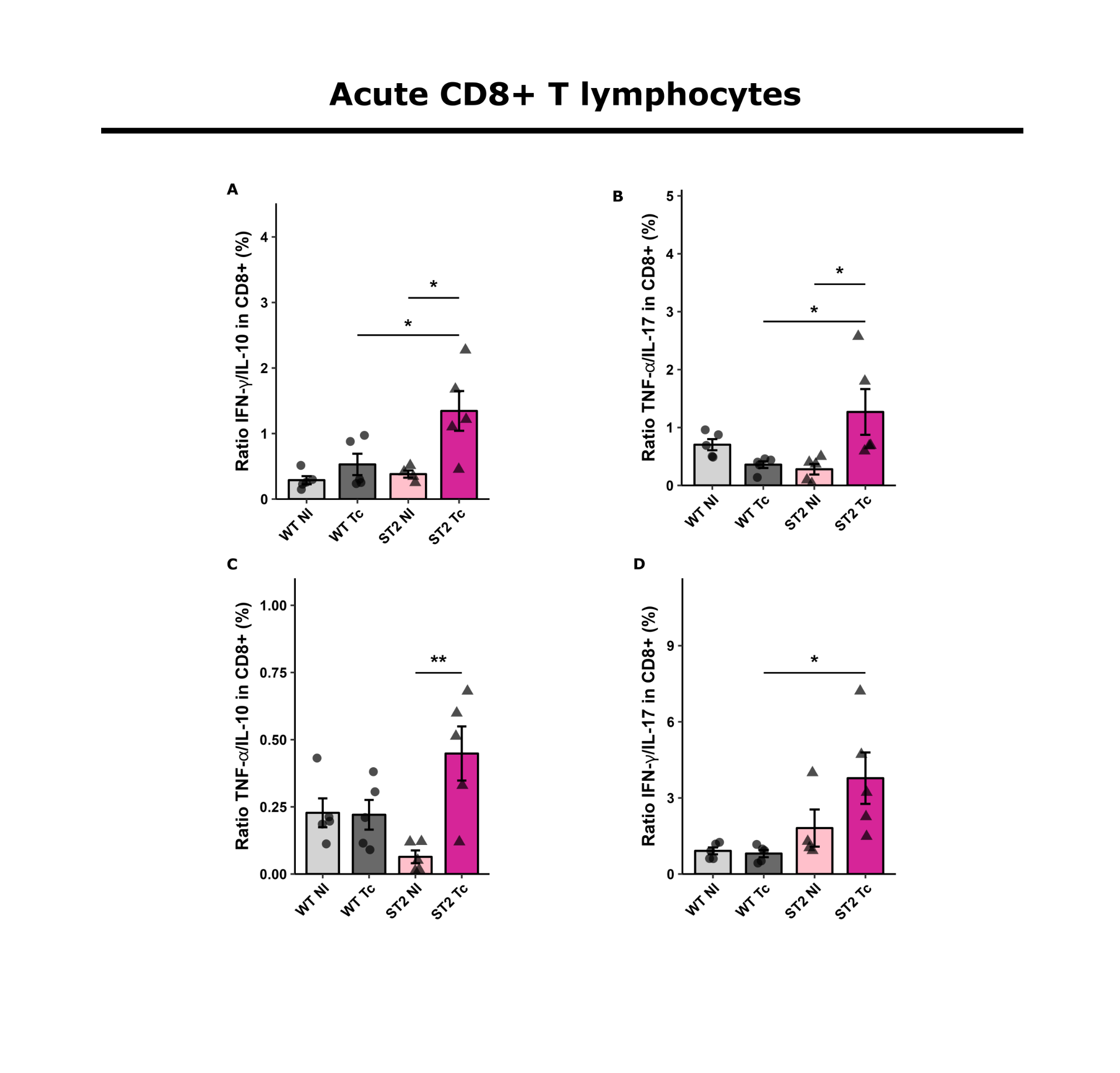
**

**Supplementary figure 7. Enhanced inflammatory effector ratios in CD8+ T cells of ST2-deficient mice during acute infection.** Analysis of cytokine production balance in CD8+ T lymphocytes. (A) IFN-γ/IL-10, (B) TNF/IL-17, (C) TNF/IL-10, and (D) IFN-γ/IL-17 ratios. Data represent mean ± SEM from N = 5–6 mice/group. *p < 0.05, **p < 0.01 (One‑way ANOVA with Tukey’s post‑hoc). WT: wild‑type; ST2^-/-^ : ST2‑deficient.

**
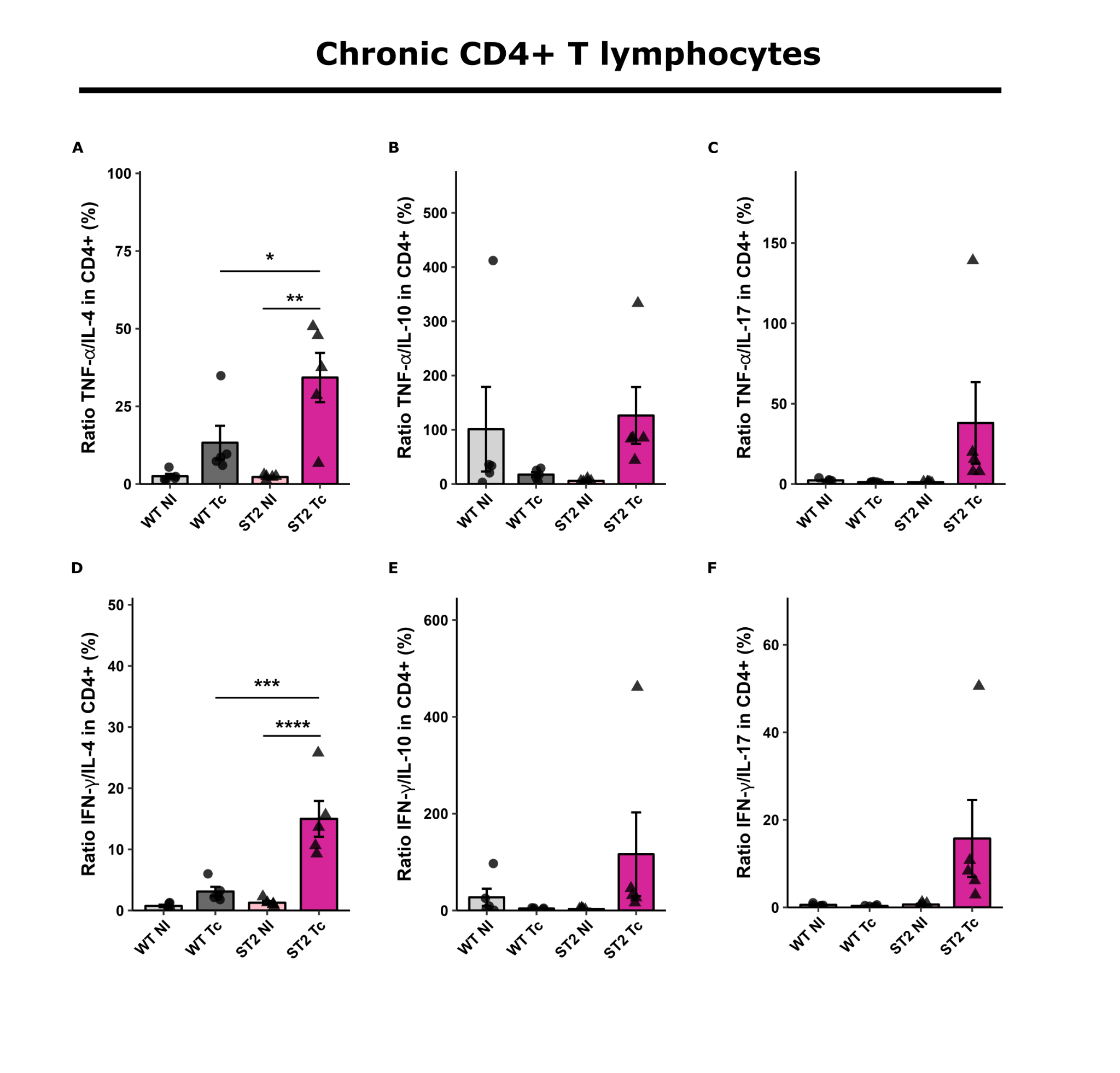
**

**Supplementary figure 8. ST2-deficiency leads to sustained and exacerbated pro-inflammatory CD4+ T cell ratios in chronic Chagas disease**. Cytokine ratios in splenic CD4+ T cells at 100 DPI. (A-C) TNF ratios and (D-F) IFN-γ ratios relative to IL-4, IL-10, and IL-17 in the chronic phase. Data represent mean ± SEM from N = 5–6 mice/group. *p < 0.05, **p < 0.01 (One‑way ANOVA with Tukey’s post‑hoc). WT: wild‑type; ST2^-/-^ : ST2‑deficient.


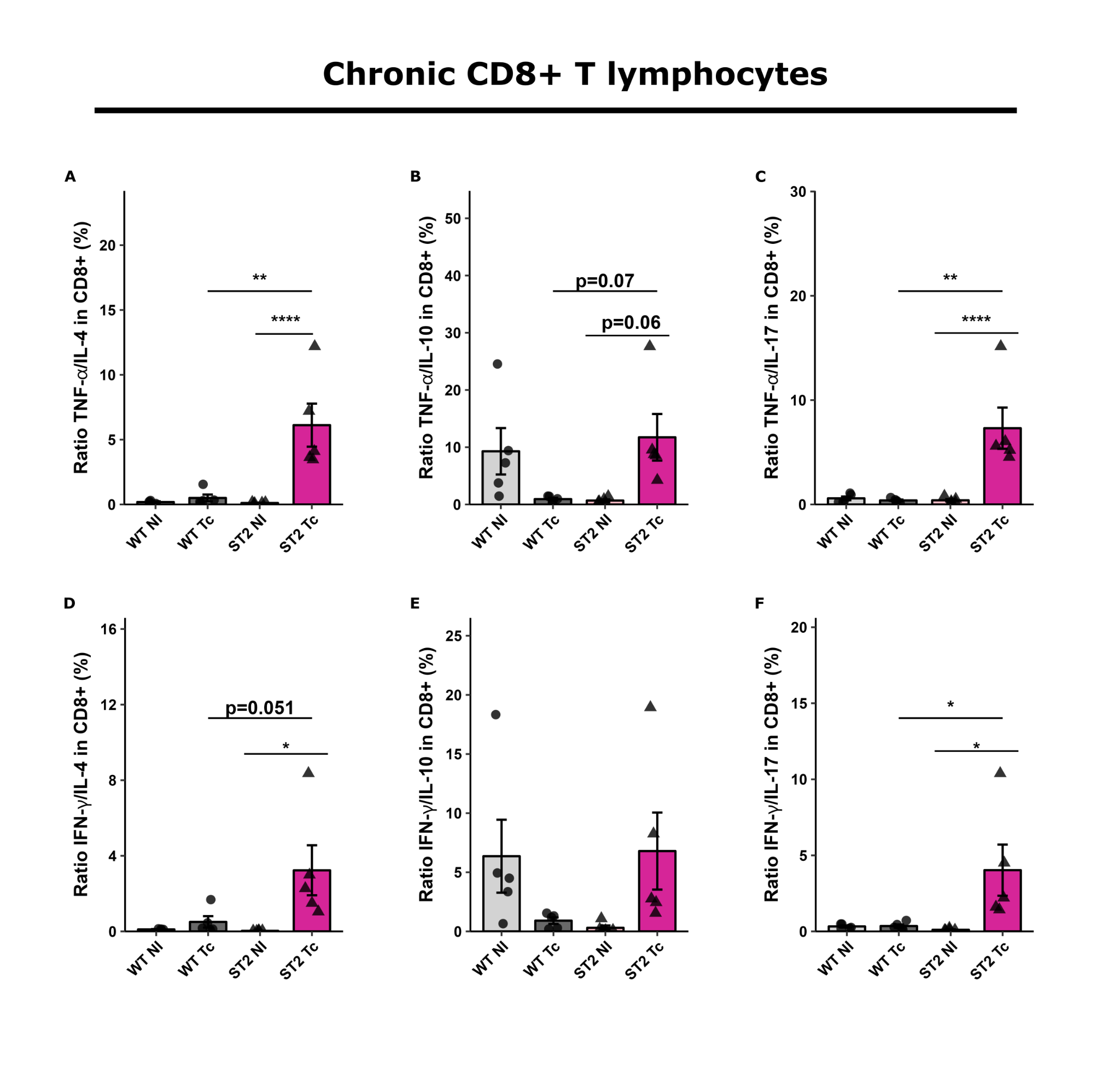


**Supplementary figure 9. Persistent Th1-like bias in CD8+ T cells of ST2-deficient mice during the chronic stage of *T. cruzi* infection.** Evaluation of CD8+ T cell cytokine ratios at 100 DPI. (A-F) Comprehensive analysis of inflammatory balance. ST2 KO mice maintain significantly higher TNF and IFN-γ ratios relative to IL-4 and IL-17 compared to WT mice. Data represent mean ± SEM from N = 5–6 mice/group. *p < 0.05, **p < 0.01 (One‑way ANOVA with Tukey’s post‑hoc). WT: wild‑type; ST2^-/-^ : ST2‑deficient.


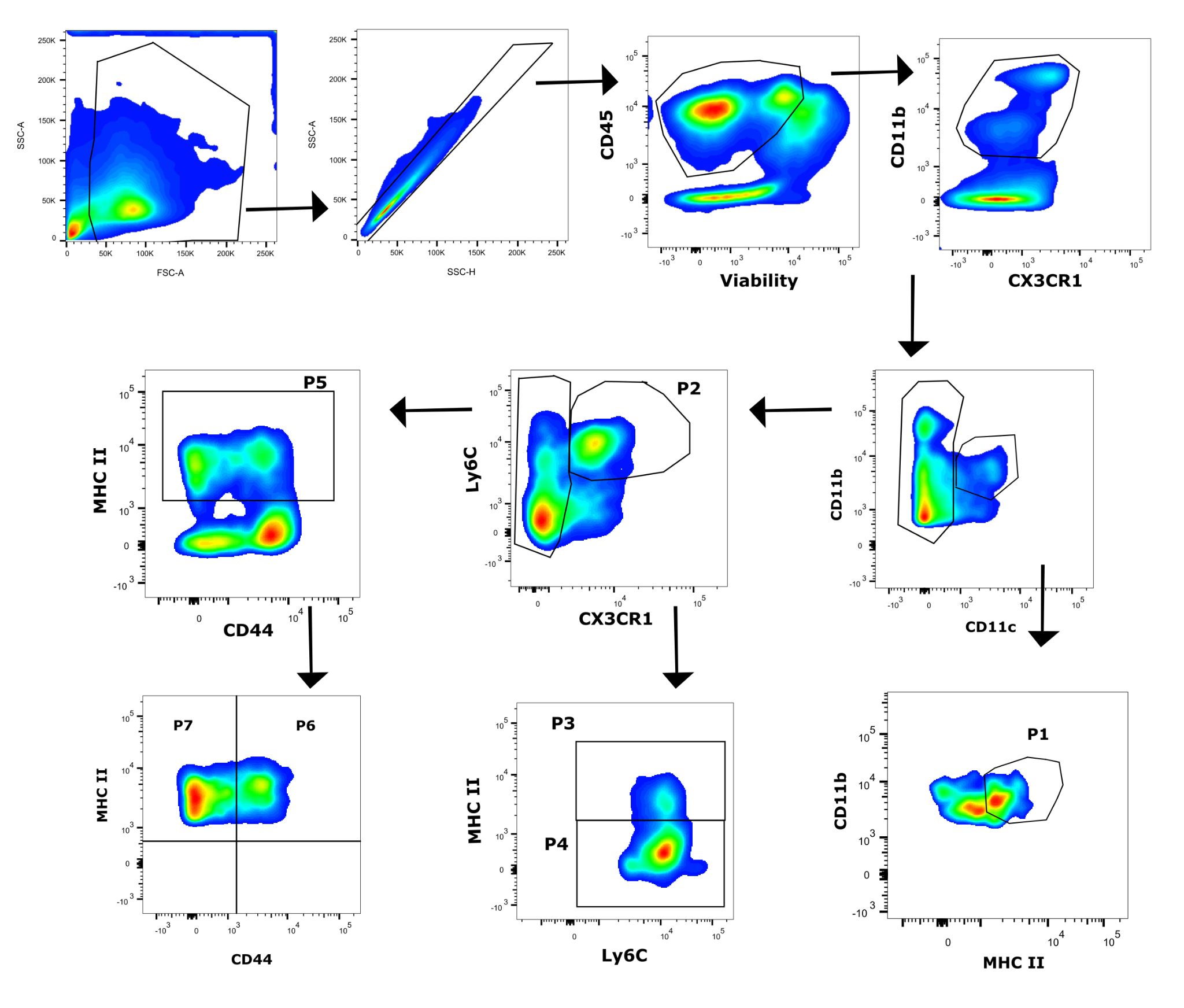


**Supplementary figure 10. Multi-parametric flow cytometry identification of monocyte and macrophage subpopulations.** Representative gating strategy for the characterization of myeloid compartments in the spleen. Total leukocytes were gated for size/granularity and singlets. CD45+ viable cells were selected for lineage analysis. CD11b+ cells were further subdivided based on CX3CR1, Ly6C, and MHC II expression to identify: P1 (dendritic cells), P2 monocytes, P3/P4 monocyte subsets (classical Ly6C^high^ and patrolling Ly6C^low^), and P5-P7 (total macrophages and subsets based on CD44 activation markers).
